# Expression of T-Bet, Eomesodermin, and GATA-3 Correlates With Distinct Phenotypes and Functional Properties in Porcine γδ T Cells

**DOI:** 10.3389/fimmu.2019.00396

**Published:** 2019-03-11

**Authors:** Irene M. Rodríguez-Gómez, Stephanie C. Talker, Tobias Käser, Maria Stadler, Lisa Reiter, Andrea Ladinig, Jemma V. Milburn, Sabine E. Hammer, Kerstin H. Mair, Armin Saalmüller, Wilhelm Gerner

**Affiliations:** ^1^Institute of Immunology, Department of Pathobiology, University of Veterinary Medicine Vienna, Vienna, Austria; ^2^University Clinic for Swine, Department for Farm Animals and Veterinary Public Health, University of Veterinary Medicine Vienna, Vienna, Austria; ^3^Christian Doppler Laboratory for Optimized Prediction of Vaccination Success in Pigs, Institute of Immunology, Department of Pathobiology, University of Veterinary Medicine Vienna, Vienna, Austria

**Keywords:** γδ T cells, swine, pig, T-bet, Eomes, GATA-3

## Abstract

Unlike mice and humans, porcine γδ T cells represent a prominent subset of T cells in blood and secondary lymphatic organs. GATA-3, T-bet and Eomesodermin (Eomes) are transcription factors with crucial functions in T-cell development and functional differentiation, but their expression has not been investigated in porcine γδ T cells so far. We analyzed the expression of these transcription factors in γδ thymocytes, mature γδ T cells from blood, spleen, lymph nodes, and lung tissue as well as *in vitro* stimulated γδ T cells on the protein level by flow cytometry. GATA-3 was present in more than 80% of all γδ-thymocytes. Extra-thymic CD2^−^ γδ T cells expressed high levels of GATA-3 in all investigated organs and had a CD8α^−/dim^CD27^+^perforin^−^ phenotype. T-bet expression was mainly found in a subset of CD2^+^ γδ T cells with an opposing CD8α^high^CD27^dim/−^perforin^+^ phenotype. Eomes^+^ γδ T cells were also found within CD2^+^ γδ T cells but were heterogeneous in regard to expression of CD8α, CD27, and perforin. Eomes^+^ γδ T cells frequently co-expressed T-bet and dominated in the spleen. During aging, CD2^−^GATA-3^+^ γδ T cells strongly prevailed in young pigs up to an age of about 2 years but declined in older animals where CD2^+^T-bet^+^ γδ T cells became more prominent. Despite high GATA-3 expression levels, IL-4 production could not be found in γδ T cells by intracellular cytokine staining. Experiments with sorted and ConA + IL-2 + IL-12 + IL-18-stimulated CD2^−^ γδ T cells showed that proliferating cells start expressing CD2 and T-bet, produce IFN-γ, but retain GATA-3 expression. In summary, our data suggest a role for GATA-3 in the development of γδ-thymocytes and in the function of peripheral CD2^−^CD8α^−/dim^CD27^+^perforin^−^ γδ T cells. In contrast, T-bet expression appears to be restricted to terminal differentiation stages of CD2^+^ γδ T cells, frequently coinciding with perforin expression. The functional relevance of high GATA-3 expression levels in extra-thymic CD2^−^ γδ T cells awaits further clarification. However, their unique phenotype suggests that they represent a thymus-derived separate lineage of γδ T cells in the pig for which currently no direct counterpart in rodents or humans has been described.

## Introduction

γδ T cells are an evolutionarily conserved subset of T cells which is present in all jawed vertebrates (1). In contrast to mice and humans, γδ T cells in swine are a prominent subset of T cells in blood but also in other secondary lymphatic organs (2–5). Despite this prominence and the long-standing research activities on porcine γδ T cells (6), knowledge on phenotypic and functional traits of this T-cell subset is still limited.

Research in murine models has shown that various transcription factors (TFs) are relevant for the development of distinct immune cell lineages and functional subsets by activating or repressing their target genes, frequently alongside the action of particular cytokines. The TF T-bet has originally been described as the master regulator of the Th1 response (7) but over the years it has become clear that T-bet is also involved in the type-1 related differentiation, function, migration, and survival of other CD4^+^ T cell subsets as well as CD8^+^ T cells, B cells and innate lymphoid cells (8). The TF Eomesodermin (Eomes) regulates the function and development of cytotoxic CD8^+^ T cells and cytotoxic CD4^+^ Th1 cells, by inducing the expression of IFN-γ, perforin, and granzyme B (9, 10). Moreover, Eomes, together with T-bet, controls the differentiation of CD8^+^ T cells into long-lived memory cells or short-lived effector cells. Whereas, Eomes fosters the development of memory CD8^+^ T cells, T-bet seems to play a pivotal role in the maintenance of short-lived effector CD8^+^ T cells (11, 12). Studies in mouse and human have shown the expression of Eomes mRNA and protein by γδ T cells (13–15). Similarly, T-bet expression was found in murine (16) and human γδ T cells (14). The TF GATA-3 is well-known for its function as a master regulator of Th2 responses (17). However, this TF is also involved in T-cell lineage commitment (18), having a pivotal role already during the transition of thymus-seeding progenitor cells into early T-lineage progenitors (19). Later during T-cell development in the thymus, GATA-3 is required for transition from the double negative T-cell stage 1 to double negative T-cell stages 2–3 (20, 21), which also includes γδ T-cell development (19). In addition, GATA-3 acts together with ThPOK as a TF for CD4-lineage commitment (22) and contributes to iNKT-cell development in the thymus (23). Beyond thymic development, GATA-3 has also been reported to be of relevance for the maintenance and survival of CD4^+^ and CD8^+^ T cells in the periphery (24) as well as Treg survival and function (25). In contrast, not much is known about the relevance of GATA-3 in mature γδ T cells; an early study with murine γδ T cells from the spleen could identify GATA-3 expression only after *in vitro* stimulation with IL-4 (16).

Despite these findings, to our knowledge the expression of GATA-3, T-bet and Eomes has not been investigated in porcine γδ T cells. Thus, we reasoned that analyzing these TFs in γδ T cells isolated from different lymphatic and non-lymphatic organs, as well as from pigs of different age, would provide a more detailed insight into potential functional and developmental properties of respective γδ T-cell subsets. We could identify prominent subpopulations of γδ T cells expressing all three TFs. In particular GATA-3 and T-bet expressing γδ T cells had largely opposing phenotypes and showed age-related changes in their relative abundance. Moreover, our data indicate that GATA-3 expression in porcine γδ T cells is not related to IL-4 production but rather seems to be a phenomenon of the CD2^−^ γδ T-cell subset. Overall, this suggests that CD2^−^ γδ T cells differ substantially from other γδ T-cell subsets, although their functional properties still await a thorough investigation.

## Materials and Methods

### Animals and Cell Isolation

Blood and organs were collected from 7-month-old finishing pigs and 4- to 5-year-old healthy sows from an abattoir. Animals were anesthetized using a high voltage electric device and thereafter exsanguinated. This procedure is in accordance to the Austrian Animal Welfare Slaughter Regulation. For analyses of peripheral blood mononuclear cells (PBMCs) in aging pigs, piglets were repeatedly sampled at 3 weeks, 25 weeks, and 26 months of age. The recurrent blood sampling of these animals was approved by the institutional ethics committee, the Advisory Committee for Animal Experiments (§12 of Law for Animal Experiments, Tierversuchsgesetz—TVG) and the Federal Ministry for Science and Research (reference number BMWF-68.205/0021-II/3b/2011).

PBMCs were obtained by gradient centrifugation with lymphocyte separation medium (density 1.077 g/mL; PAN Biotech, Aidenbach, Germany) as described previously (26). Lymphocytes from thymus, spleen, mediastinal lymph node and lung tissue were isolated as reported previously (27, 28). Isolated lymphocytes were either processed for immediate analysis by flow cytometry (FCM), or cultivated *in vitro* (see details below). For some experiments, PBMCs were initially frozen at −150°C following a previously described procedure (29).

### Fluorescence-Activated Cell Sorting (FACS)

For sorting of total γδ T cells and CD2^−^ γδ T cells, defrosted PBMCs were used. Up to 2 × 10^8^ PBMCs were re-suspended in 500 μL of sorting medium consisting of RPMI 1640 supplemented with 5% (v/v) heat-inactivated fetal calf serum (FCS) (both from PAN Biotech) and 5% (v/v) heat-inactivated porcine plasma (in house preparation) and 2 mM EDTA. PBMCs were labeled with primary monoclonal antibodies (mAbs) against TCR-γδ (clone PGBL22A, mouse IgG1, VMRD, Pullman, WA, USA) and CD2 (clone MSA4, mouse IgG2a, in house). Cells were washed in sorting medium, re-suspended, and incubated with second-step reagents: rat anti-mouse IgG1-PerCP (BD Biosciences, San Jose, CA, USA) and goat anti-mouse IgG2a-Alexa488 (Thermo Fisher, Waltham, MA, USA). After two further washing steps, cells were sorted using a FACSAria cell sorter (BD Biosciences). The purity of sorted cell populations varied from 99.3 to 99.6 for total γδ T cells (mean of 99.5%) and from 99.7 to 99.9 for CD2^−^ γδ T cells (mean of 99.8%).

### *In vitro* Cultivation and FCM Analysis of Sorted γδ T Cells

For parallel analysis of proliferation and phenotype, sorted γδ T cells and sorted CD2^−^ γδ T cells as well as total PBMCs were labeled using the CellTrace™ Violet Cell Proliferation Kit (Thermo Fisher) prior to cultivation as described elsewhere (27). Subsequently, 2 × 10^5^ cells per well were cultivated for 4 days at 37°C in round-bottomed 96-well plates (Greiner Bio-One, Kremsmünster, Austria), under the following conditions: (i) unstimulated in cell culture medium (RPMI 1640 with stable glutamine supplemented with 10% (v/v) heat-inactivated FCS, 100 IU/mL penicillin and 0.1 mg/mL streptomycin, all from PAN Biotech), (ii) Concanavalin A (ConA) (5 μg/mL, Amersham Biosciences, Uppsala, Sweden) combined with recombinant porcine (rp) IL-2 (20 ng/mL, R&D Systems, Minneapolis, MN, USA) or (iii) ConA in combination with rpIL-2, rpIL-12 and rpIL-18 (5 μg/mL, 20 ng/mL, 25 ng/mL, and 25 ng/mL, respectively; R&D Systems, Minneapolis, MN, USA) in a final volume of 200 μL. After 4 days of cultivation, cells were harvested, re-stained for TCR-γδ and CD2 expression using the same primary and secondary antibodies mentioned above and, following fixation and permeabilization, stained with mAbs for T-bet, Eomes and GATA-3. Details on the staining procedure are described in chapter 2.5 and antibodies used are listed in Table 1. In parallel to the harvest of the cells, supernatants of the microcultures were collected and stored at −80°C for analysis of cytokines.

**Table 1:** Antibody panels used for FCM.

**Antigen**	**Clone**	**Isotype**	**Fluorochrome**	**Labeling strategy**	**Source of primary Ab**
**THYMOCYTES**					
TCR-γδ	PGBL22A	IgG1	Alexa647	Secondary antibody^b^	VMRD
CD2	MSA4	IgG2a	Alexa488	Directly conjugated	In house
CD8α	11/295/33	IgG2a	BV421	Two step biotin-streptavidin^c^	In house
T-bet	eBio4B10	IgG1	PE	Directly conjugated	eBioscience
Eomes	WD1928	IgG1	PE^a^	Directly conjugated	eBioscience
GATA-3	TWAJ	IgG2b	PE^a^	Directly conjugated	eBioscience
**γ*δ* T CELLS FROM BLOOD, SPLEEN, mLN AND LUNG**					
TCR-γδ	PPT-16	IgG2b	BV421	Two step biotin-streptavidin^d^	In house
CD2	MSA4	IgG2a	Alexa488	Directly conjugated	In house
CD8α	11/295/33	IgG2a	PE-Cy7	Secondary antibody^e^	In house
CD27	b30c7	IgG1	Alexa647	Secondary antibody^b^	In house
Perforin	δ-G9	IgG2b	PerCP-eFluor710	Directly conjugated	eBioscience
T-bet	eBio4B10	IgG1	PE	Directly conjugated	eBioscience
Eomes	WD1928	IgG1	PE^a^	Directly conjugated	eBioscience
GATA-3	TWAJ	IgG2b	PE^a^	Directly conjugated	eBioscience
**CO-EXPRESSION OF T-BET AND EOMES ON T CELLS DERIVED FROM BLOOD, SPLEEN AND LUNG**					
TCR-γδ	PPT-16	IgG2b	PE-Cy7	Two step biotin-streptavidin^f^	In house
CD2	MSA4	IgG2a	Alexa488	Secondary antibody^g^	In house
CD27	b30c7	IgG1	BV421	Secondary antibody^h^	In house
T-bet	eBio4B10	IgG1	eFluor660	Directly conjugated	eBioscience
Eomes	WD1928	IgG1	PE	Directly conjugated	eBioscience
**ANALYSIS OF IL-4 PRODUCTION BY ICS**					
CD3	PPT3	IgG1	Alexa488	Secondary antibody^i^	In house
CD4	b38c6c7	IgG1	Alexa488^a^	Secondary antibody^i^	In house
TCR-γδ	PGBL22A	IgG1	Alexa488^a^	Secondary antibody^i^	VMRD
CD8α	11/295/33	IgG2a	PE	Secondary antibody^j^	In house
IL-4	A155B16F2	IgG2b	Alexa647	Secondary antibody^k^	Thermo Fisher
**ANALYSIS OF FACS-SORTED γ*δ* T CELLS**					
TCR-γδ	PGBL22A	IgG1	PerCP	Secondary antibody^l^	VMRD
CD2	MSA4	IgG2a	Alexa488	Secondary antibody^g^	In house
T-bet	eBio4B10	IgG1	eFluor660	Directly conjugated	eBioscience
Eomes	WD1928	IgG1	PE^a^	Directly conjugated	eBioscience
GATA-3	TWAJ	IgG2b	PE^a^	Directly conjugated	eBioscience

a *Used in parallel samples*.

b *Goat anti-Mouse IgG1-Alexa647, Thermo Fisher*.

c *Goat anti-Mouse IgG2a-biot., Southern Biotech; Streptavidin-BV421, BioLegend*.

d *Goat anti-Mouse IgG2b-biot., Southern Biotech; Streptavidin-BV421, BioLegend*.

e *Goat anti-Mouse IgG2a-PE-Cy7, Southern Biotech*.

f *Goat anti-Mouse IgG2b-biot., Southern Biotech; Streptavidin-PE-Cy7, eBioscience*.

g *Goat anti-Mouse IgG2a-Alexa488, Thermo Fisher*.

h *Rat anti-Mouse IgG1-BV421, BioLegend*.

i *Goat anti-Mouse IgG1-Alexa488, Thermo Fisher*.

j *Goat anti-Mouse IgG2a-PE, Southern Biotech*.

k *Goat anti-Mouse IgG2b-Alexa647, Thermo Fisher*.

l *Rat anti-Mouse IgG1-PerCP, BD Biosciences*.

### Intracellular Cytokine Staining for IL-4

Defrosted PBMCs were placed in cell culture medium overnight with 5 × 10^5^ cells per well at 37 °C in round-bottomed 96-well plates. On the following morning, cells were stimulated for 4 h with phorbol 12-myristate 13-acetate (PMA, 50 ng/mL, Sigma-Aldrich, Schnelldorf, Germany) and ionomycin (500 ng/mL, Sigma-Aldrich) combined with Brefeldin A (1 μg/mL, BD GolgiPlug™, BD Biosciences). Cells were harvested and stained for CD3, CD4, TCR-γδ, and CD8α, followed by intracellular cytokine staining (ICS) for IL-4. Details on the staining procedure are described in the following chapter and antibodies used are listed in Table 1.

### FCM Staining

Table 1 provides technical information on mAbs and secondary reagents. Incubation with mAbs specific for extracellular antigens was performed for 20 min in the fridge in round-bottomed 96-well plates. After incubation with primary and secondary reagents, the cells were washed two times with 200 μL PBS + 10% (v/v) porcine plasma (in house preparation). For *in vitro* cultivated cells, PBS containing 3% (v/v) FCS was used for washing. After labeling of antigens in the cell membrane, samples were fixed and permeabilized with Foxp3/Transcription Factor Staining Buffer Set (eBioscience, San Diego, CA, USA) following the manufacturer's instructions. For samples where IL-4 was analyzed by ICS, fixation and permeabilization was performed with BD Cytofix/Cytoperm and BD Perm/Wash (both BD Biosciences), respectively. For intracellular staining of TFs, single directly conjugated mAbs or mastermixes of directly conjugated antibodies were used and incubated for 30 min in the fridge. Intracellular IL-4 was labeled by a two-step procedure with a porcine IL-4-specific primary antibody followed by a second incubation with a fluorochrome-labeled isotype-specific secondary antibody. Between the intracellular incubation steps, cells were washed twice with BD Perm/Wash.

For samples where TF expression was analyzed in combination with isotype-specific secondary antibodies for cell surface labeling, whole mouse IgG molecules (1 μg per sample; ChromPure; Jackson ImmunoResearch, West Grove, PA, USA) were added after the second incubation step to block potentially free binding sites of secondary antibodies. This was done in a separate incubation step before subsequent incubation with directly-conjugated mAbs. During this incubation with IgG molecules, a staining with Live/Dead® Near-IR (Thermo Fisher) was performed according to the manufacturer's instructions. For samples used for IL-4 analysis, Fixable Viability Dye eFluor 780 (eBioscience) was used during incubation with secondary antibodies for cell surface markers. For the staining panels listed in Table 1, isotype-matched control samples were prepared to check for unspecific binding. Samples stained with single antibodies were prepared for calculation of compensation and, where appropriate, fluorescence minus one (FMO) samples were prepared.

For a bulk staining of non-γδ T cells, PBMCs isolated from 7-months-old pigs were labeled with antibodies against CD4 (clone b38c6c7), CD8β (clone PPT23), CD16 (clone G7), and CD172a (clone 74-22-15). These mAbs have a mouse IgG1 isotype and were produced in house, with the exception of G7, which was purchased from Bio-Rad (Puchheim, Germany). Subsequently, the mAbs were labeled with a goat anti-mouse IgG1-Alexa647 secondary antibody (Table 1, footnote “b”) and in a third incubation step Fixable Viability Dye eFluor 780 and whole mouse IgG were added to the samples. Following fixation and permeabilization with the Foxp3/Transcription Factor Staining Buffer Set (see above), samples were further labeled with mAbs against CD79α (clone HM57, conjugated with Alexa647, Agilent-Dako) and GATA-3 (clone TWAJ, PE-conjugated, see Table 1 for details). During primary incubation, some samples were additionally stained with mAbs against TCR-γδ (clone PPT16, further labeled with goat anti-mouse IgG2b-BV421 (Jackson ImmunoResearch, Ely, United Kingdom) and CD2 (clone MSA4, further labeled with goat anti-mouse IgG2a-Alexa488, Table 1, footnote “g”).

### FCM Analysis

FCM samples were analyzed on a FACSCanto II (BD Biosciences) flow cytometer with three lasers (405, 488, and 633 nm). For samples addressing TF expression in combination with other markers, at least 1 × 10^5^ lymphocytes per sample were recorded. For samples analyzing IL-4 production, between 5 × 10^5^ and 1 × 10^6^ lymphocytes per sample were recorded. In the case of violet proliferation assays where TCR-γδ, CD2 and TF expression were analyzed, around 1.2 × 10^5^ cells per sample were recorded. Compensation was calculated by FACSDiva software version 6.1.3 (BD Biosciences) after analysis of single-stained samples. Data were further analyzed by FlowJo software, version 10.4.2. For all phenotypic analyses performed, cells were gated according to light scatter properties (FSC-A vs. SSC-A) and subjected to doublet (FSC-H vs. FSC-W and SSC-H vs. SSC-W) and dead cell discrimination, as described elsewhere (28). Only in the case of proliferation assays doublet discrimination was not carried out.

### Cytokine Quantification by Multiplex Fluorescent Microsphere Immunoassay (FMIA)

Supernatants of FACS-sorted and cultivated γδ T cells, CD2^−^ γδ T cells, and PBMCs (see chapters 2.2 and 2.3) were analyzed for the presence of IFN-γ and IL-4 by a multiplex fluorescent microsphere immunoassay (FMIA) as described elsewhere (30). The following reagents from Thermo Fisher were used in this assay: IL-4 capture and detection antibody pair, catalog numbers CSC1283 part 5S.128.09 and ASC0849, IL-4 standard CSC1283 part 5S.128.10; IFN-γ capture and detection antibody pair, catalog numbers MP700 and MP701B, IFN-γ standard CSC4033 part SD066.

### Statistical Analysis

For statistical analysis of data, IBM® SPSS® Statistics program was used (SPSS Statistics Version 24.0, IBM Corp., Armonk, NY, USA). Data sets were verified for normal distribution by the Shapiro-Wilk's test and equality of variances for the groups was assessed by Levene's test. Where necessary, data sets were subjected to log-transformation to meet these criteria. Data sets fulfilling both were analyzed by one-way variance analysis for independent samples with Bonferroni correction for paired samples. Otherwise, data sets were analyzed by the non-parametric Kruskal–Walli's test, with Bonferroni correction as *post-hoc* analysis. Three levels of significance were defined: *p* ≤ 0.05 (^*^), *p* ≤ 0.01 (^**^) and *p* ≤ 0.001 (^***^).

## Results

### Expression of T-Bet, Eomes, and GATA-3 in γδ Thymocytes

**Figure 1:**
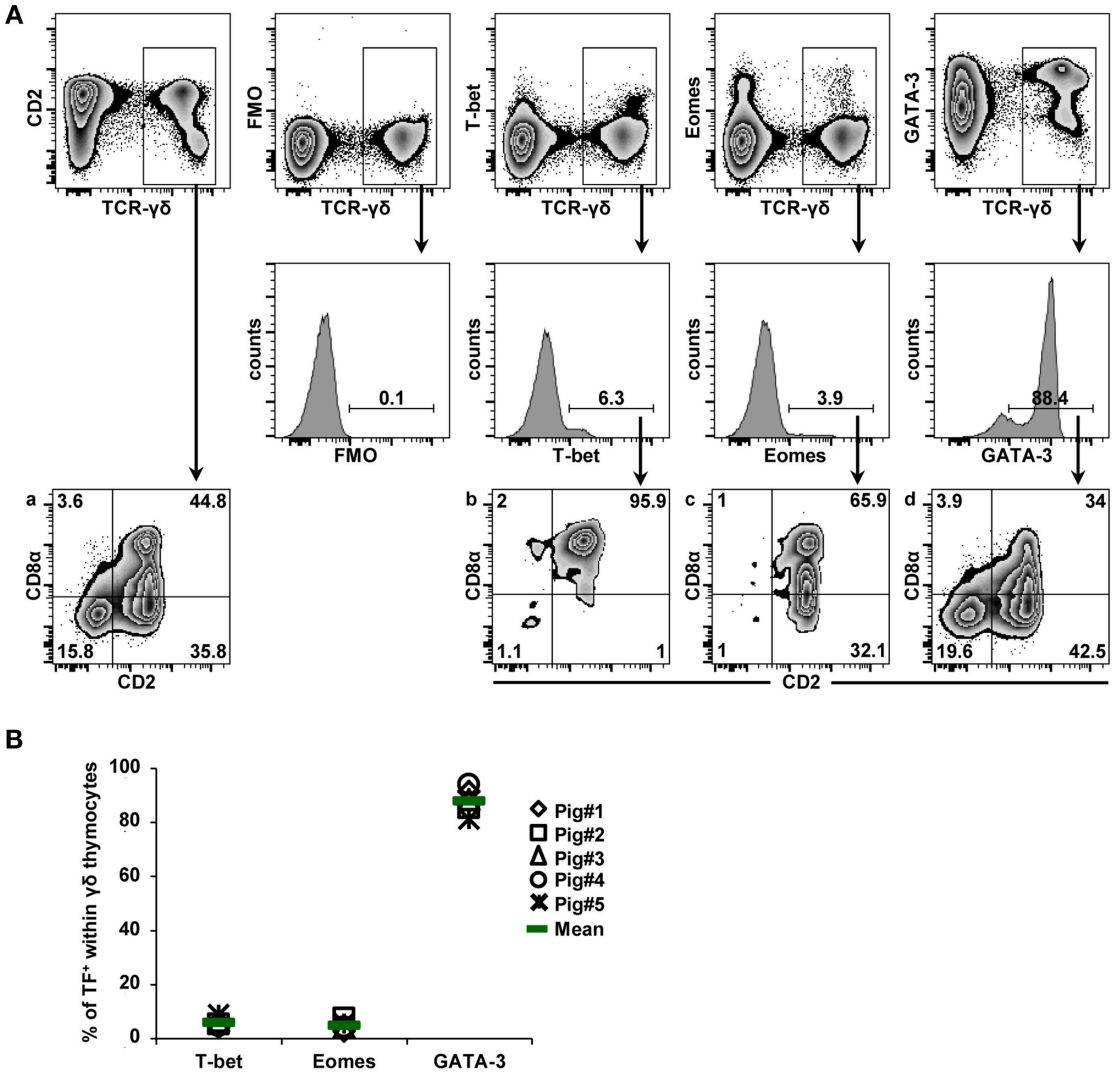
Expression of T-bet, Eomes and GATA-3 in γδ thymocytes. **(A)** Following exclusion of dead cells (not shown), total TCR-γδ^+^ thymocytes were gated (top row) and analyzed for the expression of T-bet, Eomes and GATA-3 (histograms, second row). Total TCR-γδ^+^ thymocytes and TF-expressing TCR-γδ^+^ thymocytes were further analyzed for expression of CD2 and CD8α (third row, zebra plots denoted “a,” “b,” “c,” “d”). Numbers indicate the percentage of transcription factor expressing γδ thymocytes (histograms) or percentage of CD2/CD8α-defined subsets (zebra plots, third row). Data from one representative animal out of five are shown. **(B)** The scatter diagram shows the frequency of T-bet, Eomes and GATA-3 expressing γδ thymocytes. Each symbol represents data of one individual animal (*n* = 5). Green bars indicate mean values.

T-bet, Eomes and GATA-3 expressing thymocytes were analyzed in 7-month-old pigs. Initially, thymocytes bearing the TCR-γδ were gated and further analyzed for the expression of TFs (Figure 1A, first and second row). Moreover, since γδ T cells in swine have been divided into three subsets on the basis of their CD2/CD8α expression, including CD2^−^CD8α^−^, CD2^+^CD8α^+^, and CD2^+^CD8α^−^ (31, 32), we also analyzed expression of these two molecules in total γδ thymocytes, T-bet^+^, Eomes^+^ or GATA-3^+^ γδ thymocytes (Figure 1A, third row). Figure 1A shows data of one representative animal and Figure 1B summarizes data of five pigs. T-bet^+^ and Eomes^+^ γδ thymocytes were scarce, with a mean frequency of 5.7 and 4.6%, respectively. In contrast, the vast majority of γδ thymocytes expressed GATA-3 (mean of 88%) (Figure 1B). The vast majority (~96%) of T-bet^+^ γδ thymocytes co-expressed CD2 and CD8α (Figure 1A, zebra plot “b”). Similarly, nearly all Eomes^+^ γδ thymocytes expressed CD2 (~98%), but approximately one third of them lacked CD8α expression (Figure 1A, zebra plot “c”). Whereas, GATA-3 expression was found in all CD2/CD8α-defined γδ thymocyte subsets, only the frequency of CD2^+^CD8α^+^ GATA-3^+^ thymocytes was somewhat lower than in total γδ thymocytes (Figure 1A, zebra plot “a” vs. “d”). Similar to what we previously found for TCR-αβ thymocytes (28), these data suggest that GATA-3 is of relevance in the maturation process of porcine γδ thymocytes. In contrast, T-bet and Eomes expression appear to be induced only in small γδ thymocyte subsets, probably during late stages of γδ thymocyte development.

### Analyses of T-Bet, Eomes and GATA-3 Expression in Extra-Thymic γδ T Cells

**Figure 2:**
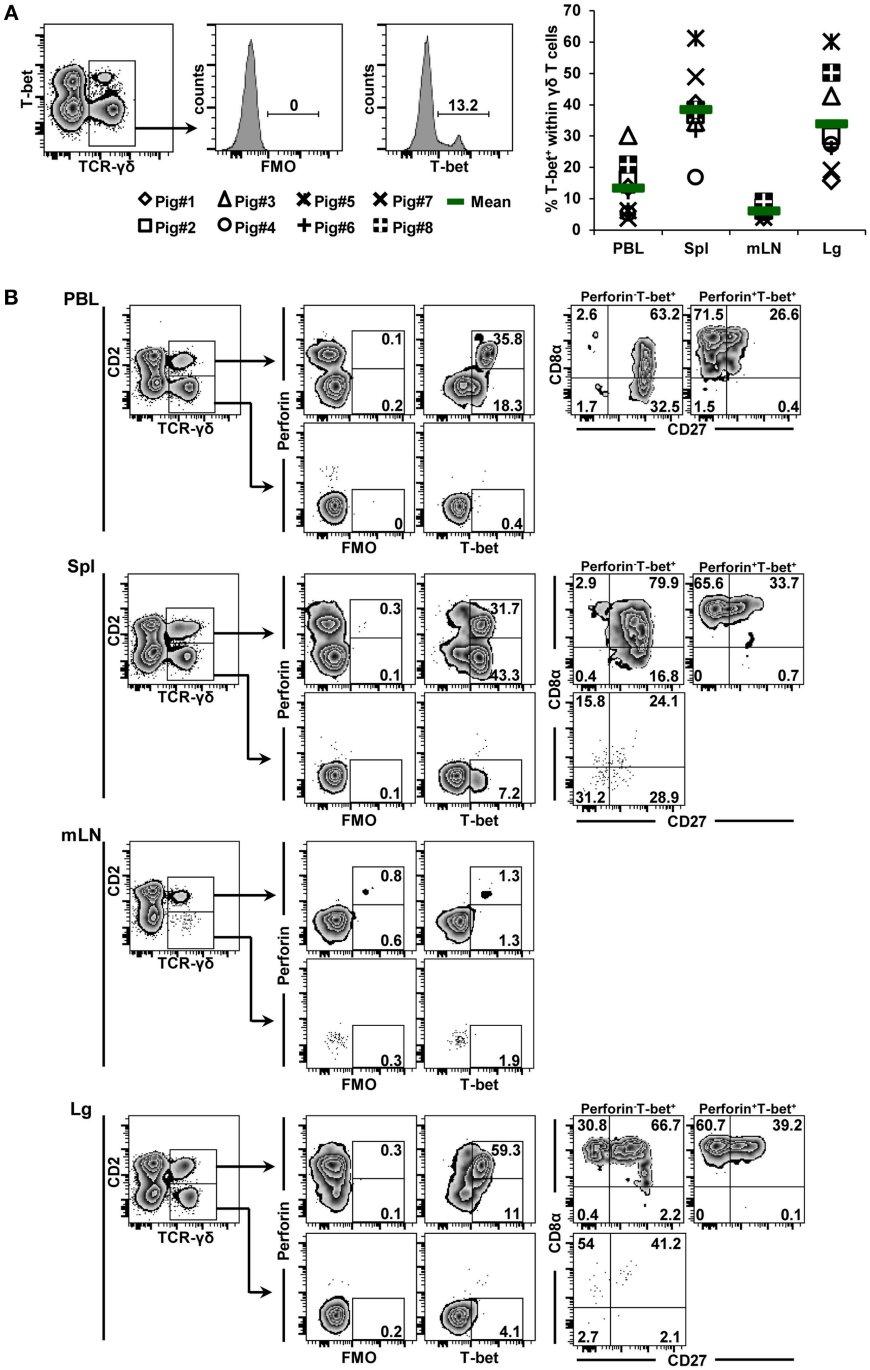
Frequency and phenotype of T-bet^+^ γδ T cells in different organs. **(A)** Following exclusion of dead cells (not shown), TCR-γδ^+^ T cells were gated within live lymphocytes (zebra plot) and further subgated to analyze the expression of T-bet. The scatter diagram shows the frequency of T-bet^+^ γδ T cells isolated from blood (peripheral blood lymphocytes, PBL), spleen (Spl), mediastinal lymph node (mLN), and lung tissue (Lg). Each symbol represents data from one individual animal (*n* = 8). Green bars indicate mean values. **(B)** γδ T cells were gated for CD2^+^ and CD2^−^ subsets and further analyzed for co-expression of perforin and T-bet. Perforin^+^ and perforin^−^ T-bet^+^ γδ T cells were then gated and investigated for expression of CD27 and CD8α. Exemplary data of the four investigated locations, blood (peripheral blood lymphocytes, PBL), spleen (Spl), mediastinal lymph node (mLN) and lung tissue (Lg) from one animal are shown. Numbers located in gates and quadrants indicate percentage of cells for one particular phenotype. For T-bet/perforin-defined subpopulations below 10%, CD8α/CD27 expression is shown in dot plots, for T-bet/perforin-defined subpopulations below 2%, CD8α/CD27 expression is not shown.

The expression of T-bet was analyzed within γδ T cells derived from blood, spleen, mediastinal lymph nodes, and lung tissue of 7-month-old pigs. γδ^+^ T-bet^+^ cells were detected in all organs (Figure 2A, scatter diagram). Mean frequencies of T-bet-expressing γδ T cells within total γδ T cells varied between organs, with spleen and lung tissue showing highest values, whereas lowest values were detected in lymph nodes (13.4, 38.4, 6.0, and 33.8% for cells isolated from blood, spleen, mediastinal lymph node and lung, respectively). We also assessed the phenotype of T-bet^+^ γδ T cells in more detail. For this purpose, we analyzed the expression of differentiation-related molecules CD2, CD8α, CD27 and perforin. Representative data of T-bet^+^ γδ T cells isolated from the different locations of one pig are displayed in the zebra plots of Figure 2B. Following separation of γδ T cells into CD2^+^ and CD2^−^ subsets, we analyzed the co-expression of perforin and T-bet. Perforin^+^ and perforin^−^ T-bet^+^ γδ T cells were then further analyzed for expression of CD27 and CD8α. Across all organs, T-bet^+^ γδ T cells were mainly found within the CD2^+^ subset of γδ T cells. Only in spleen and lung, small populations (<8%) of CD2^−^T-bet^+^ γδ T cells were found. Among CD2^+^ γδ T cells, up to 70% expressed T-bet (spleen, lung) and considerable portions of these cells co-expressed perforin, with highest frequencies present in lung tissue. Of note, perforin^+^ γδ T cells in blood and lung tissue expressed slightly higher levels of T-bet than perforin^−^ cells. Regarding CD8α/CD27 expression, CD2^+^perforin^+^T-bet^+^ γδ T cells had—across all organs—mainly a CD8α^+^CD27^−^ phenotype (>60%) followed by a CD8α^+^CD27^+^ phenotype. CD8α^−^ phenotypes were hardly present among CD2^+^perforin^+^T-bet^+^ γδ T cells. CD2^+^perforin^−^T-bet^+^ γδ T cells were mainly CD8α^+^CD27^+^ (>63%).

**Figure 3:**
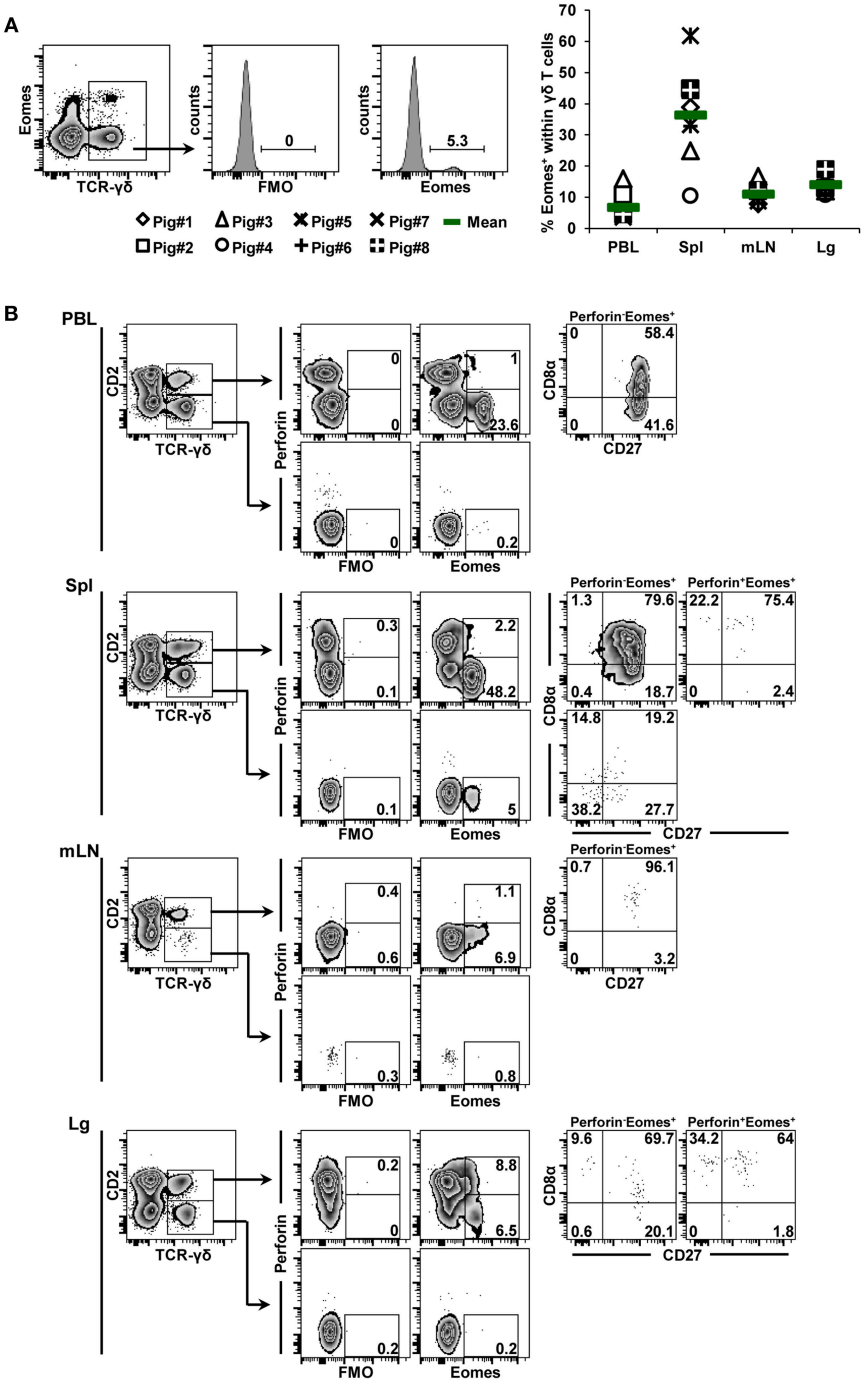
Frequency and phenotype of Eomes^+^ γδ T cells in different organs. **(A)** Following exclusion of dead cells (not shown), TCR-γδ^+^ T cells were gated within live lymphocytes (zebra plot) and further subgated to analyze the expression of Eomes. The scatter diagram shows the frequency of Eomes^+^ γδ T cells isolated from blood (peripheral blood lymphocytes, PBL), spleen (Spl), mediastinal lymph node (mLN) and lung tissue (Lg). Each symbol represents data from one individual animal (*n* = 8). Green bars indicate mean values. **(B)** γδ T cells were gated for CD2^+^ and CD2^−^ subsets and further analyzed for co-expression of perforin and Eomes. Perforin^+^ and perforin^−^ Eomes^+^ γδ T cells were then gated and investigated for expression of CD27 and CD8α. Exemplary data of the four investigated locations, blood (peripheral blood lymphocytes, PBL), spleen (Spl), mediastinal lymph node (mLN), and lung tissue (Lg) from one animal are shown. Numbers located in gates and quadrants indicate percentage of cells for one particular phenotype. For Eomes/perforin-defined subpopulations below 10%, CD8α/CD27 expression is shown in dot plots, for Eomes/perforin-defined subpopulations below 2%, CD8α/CD27 expression is not shown.

Eomes-expressing γδ T cells were also identified in all organs, with mean frequencies of 6.8, 36.4, 11.1, and 14.0% from cells of blood, spleen, mediastinal lymph node and lung tissue origin, respectively (Figure 3A, scatter diagram). The spleen was enriched for γδ^+^ Eomes^+^ cells, although the frequency of positive cells varied considerably between individual animals. In regard to CD2/perforin/CD8α/CD27 phenotypes, Eomes^+^ γδ T cells were mainly CD2^+^. Only in the spleen a minor population of CD2^−^ Eomes^+^ γδ T cells was found (5%, Figure 3B). In contrast to T-bet^+^ γδ T cells, Eomes^+^ γδ T cells were mainly perforin^−^, only in lung tissue this was different. In regard to CD8α/CD27 expression, the majority of CD2^+^perforin^−^Eomes^+^ γδ T cells had a CD8α^+^CD27^+^ phenotype (> 58%), followed by a CD8α^−^CD27^+^ phenotype. Among the few CD2^+^perforin^+^Eomes^+^ γδ T cells, also CD8α^+^CD27^−^ cells were present.

**Figure 4:**
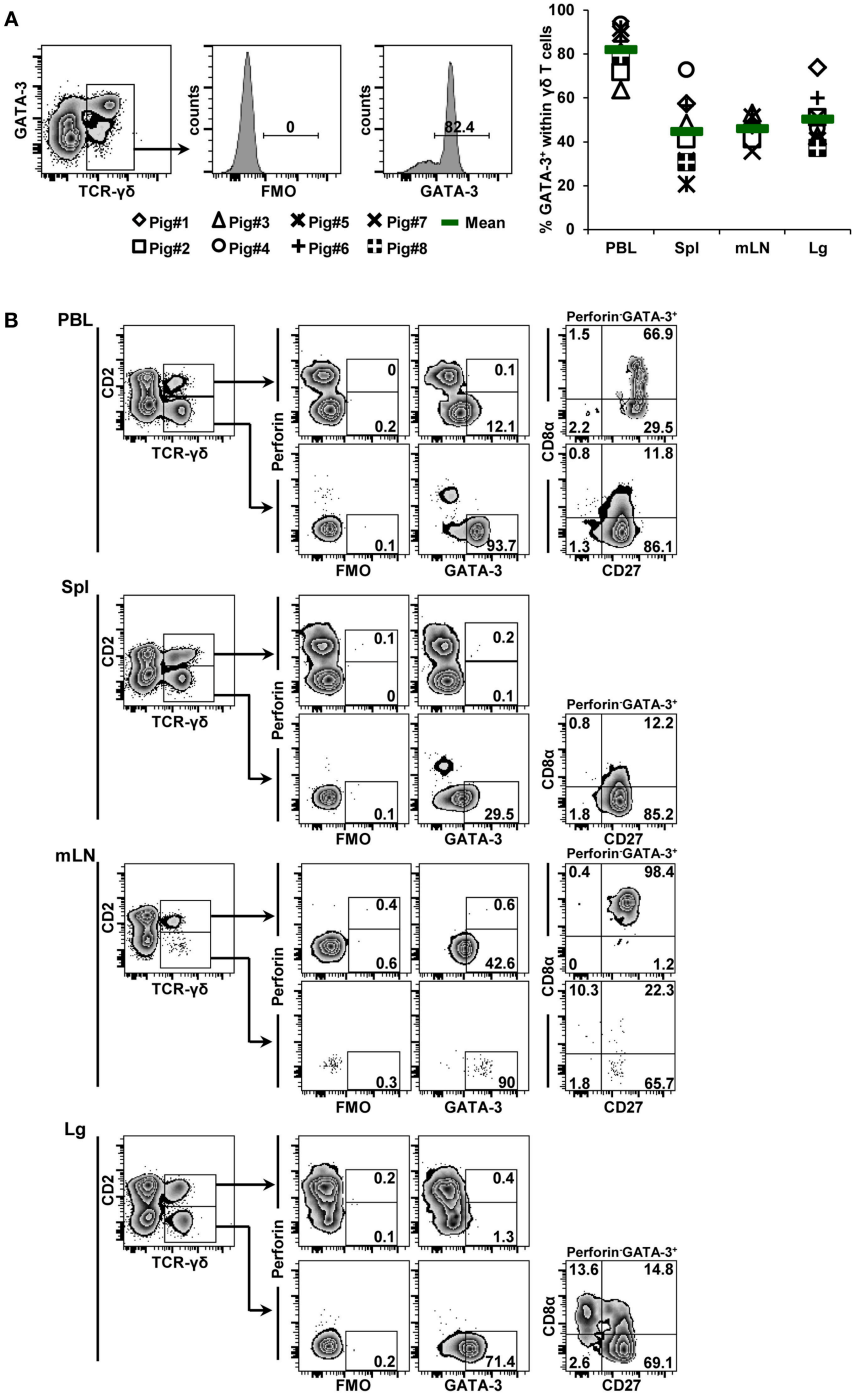
Frequency and phenotype of GATA-3^+^ γδ T cells in different organs. **(A)** Following exclusion of dead cells (not shown), TCR-γδ^+^ T cells were gated within live lymphocytes (zebra plot) and further subgated to analyze the expression of GATA-3. The scatter diagram shows the frequency of GATA-3^+^ γδ T cells isolated from blood (peripheral blood lymphocytes, PBL), spleen (Spl), mediastinal lymph node (mLN) and lung tissue (Lg). Each symbol represents data from one individual animal (*n* = 8). Green bars indicate mean values. **(B)** γδ T cells were gated for CD2^+^ and CD2^−^ subsets and further analyzed for co-expression of perforin and GATA-3. Perforin^+^ and perforin^−^ GATA-3^+^ γδ T cells were then gated and investigated for expression of CD27 and CD8α. Exemplary data of the four investigated locations, blood (peripheral blood lymphocytes, PBL), spleen (Spl), mediastinal lymph node (mLN) and lung tissue (Lg) from one animal are shown. Numbers located in gates and quadrants indicate percentage of cells for one particular phenotype. For GATA-3/perforin-defined subpopulations below 10%, CD8α/CD27 expression is shown in dot plots, for GATA-3/perforin-defined subpopulations below 2%, CD8α/CD27 expression is not shown.

GATA-3 expression was also analyzed with the same experimental set-up. Again, GATA-3^+^ γδ T cells were detected in all investigated organs (Figure 4A, scatter diagram) and mean frequency values were higher (82,0% blood; 44,8% spleen; 46.1% mediastinal lymph node; 50.2% lung) compared to both T-bet^+^ γδ T cells and Eomes^+^ γδ T cells, albeit with a considerable degree of variation between animals. Analyses on CD2, CD8α, CD27, and perforin expression showed that the majority of GATA-3^+^ γδ T cells were present in the CD2^−^ subset (Figure 4B). Of note, the overall expression level of GATA-3 varied between individual organs, since within the CD2^+^ γδ T cells some GATA-3^+^ cells were also present. Nevertheless, perforin^+^ γδ T cells present in PBL, spleen and lung tissue were clearly GATA-3^−^. In regard to CD8α/CD27 expression, the majority of CD2^−^GATA-3^+^ γδ T cells was CD8α^−^CD27^+^ (>65%), whereas the CD2^+^GATA-3^dim^ γδ T cells were mainly CD8α^+^CD27^+^. Since GATA-3 expression can be influenced by TCR-stimulation (33), we aimed to exclude the possibility that the labeling strategy by anti-TCR-γδ mAbs influenced GATA-3 expression. Therefore, PBMCs were stained with mAbs against CD4, CD8β, CD16, CD172a, and CD79α, all fluorescently labeled with Alexa647; with the aim of labeling all cells present within PBMCs with the exception of γδ T cells. In addition, samples were stained with mAbs against GATA-3, conjugated with PE. In comparison to FMO controls, a clear population of GATA-3^+^ PBMCs with a bulk-stain negative phenotype could be identified (Supplementary Figure 1, left panel). In parallel samples, which were stained for TCR-γδ and CD2 expression, the frequency of GATA-3^+^bulk-stain-negative PBMCs stayed approximately the same (Supplementary Figure 1, right panel); hence, there was no indication that a co-labeling of TCR-γδ and CD2 by mAbs influenced GATA-3 expression levels in our experimental system. When GATA-3^+^bulk-stain-negative PBMCs were gated and analyzed for CD2 and TCR-γδ expression the vast majority of cells were TCR-γδ^+^CD2^−^, confirming the phenotypes described in Figure 4B. In summary, the analyses of extra-thymic γδ T cells indicated that T-bet^+^ and GATA-3^+^ γδ T cells had rather opposing phenotypes for CD2, CD8α, CD27, and perforin, whereas Eomes^+^ γδ T cells displayed an intermediate phenotype in-between T-bet^+^ and GATA-3^+^ γδ T cells for these four molecules.

### Co-expression of T-Bet and Eomes Within γδ T Cells

**Figure 5:**
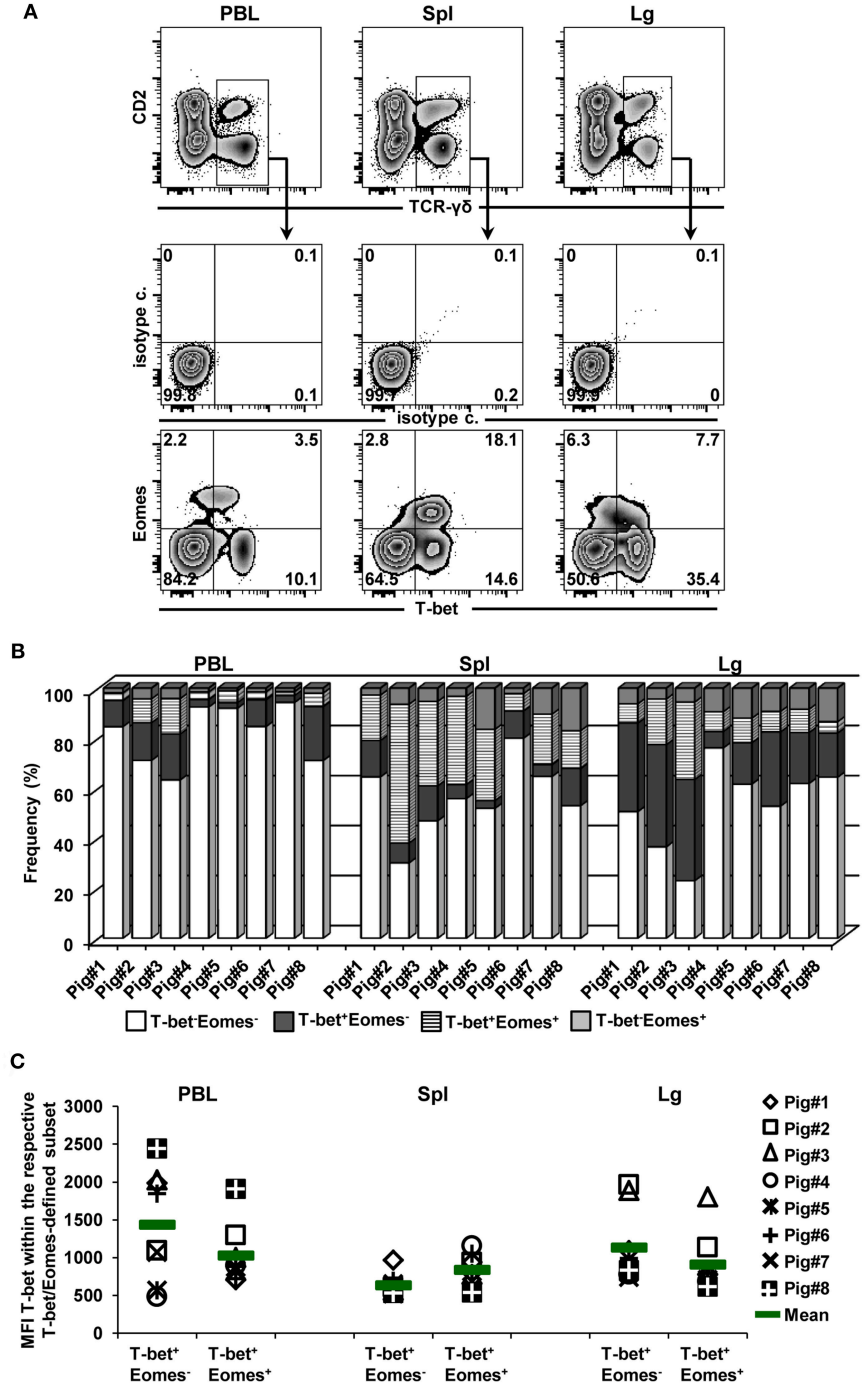
Co-expression of T-bet and Eomes within γδ T cells from different organs. **(A)** TCR-γδ^+^ T cells from blood (PBL), spleen (Spl), and lung tissue (Lg) were gated within live lymphocytes (not shown) and further analyzed for co-expression of the transcription factors T-bet and Eomes. Data from one representative animal (*n* = 8) are shown. Numbers located in quadrants indicate percentage of cells for one particular phenotype. **(B)** Stacked bars show frequencies of T-bet^−^Eomes^−^, T-bet^+^Eomes^−^, T-bet^+^Eomes^+^ and T-bet^−^Eomes^+^ γδ T cells present in PBL, spleen and lung tissue of eight different pigs. **(C)** Median fluorescence intensity (MFI) of T-bet expression in T-bet^+^Eomes^+^ and T-bet^+^Eomes^−^ γδ T-cell populations in PBL, spleen and lung tissue. Each symbol represents data from one individual animal (*n* = 8). Green bars indicate mean values.

Co-expression of the TFs T-bet and Eomes within γδ T cells isolated from the blood of humans as well as from the spleen of mice has been reported previously (14, 15). High frequencies of T-bet^+^ and Eomes^+^ γδ T cells in the spleen, together with some overlap in regard to CD2, CD8α, and CD27 expression (Figures 2, 3) suggested that within porcine γδ T cells T-bet/Eomes co-expressing cells may also exist. Hence, co-expression of both TFs within γδ T cells isolated from blood, spleen and lung tissue was investigated (Figure 5). Across these three organs, T-bet/Eomes co-expressing γδ T cells were present, but highest proportions were found in the spleen (up to 55%, but with a high animal-to-animal variation, Figures 5A,B). Initial analyses suggested that Eomes^+^T-bet^+^ γδ T cells had lower expression levels of T-bet than T-bet-single positive γδ T cells (Figure 4A, PBL and lung tissue). Indeed, on average this was the case for γδ T cells in PBL and lung from all investigated animals, but not for splenic γδ T cells (Figure 5C). Considering the coincidence of a T-bet^high^ phenotype with perforin expression (Figure 2B, PBL and lung tissue), this may suggest that T-bet^high^Eomes^−^perforin^+^ γδ T cells are in a more terminal stage of differentiation, since they also mainly had a CD27^−^ phenotype.

### Influence of Aging on Transcription Factor Expression in γδ T Cells

**Figure 6:**
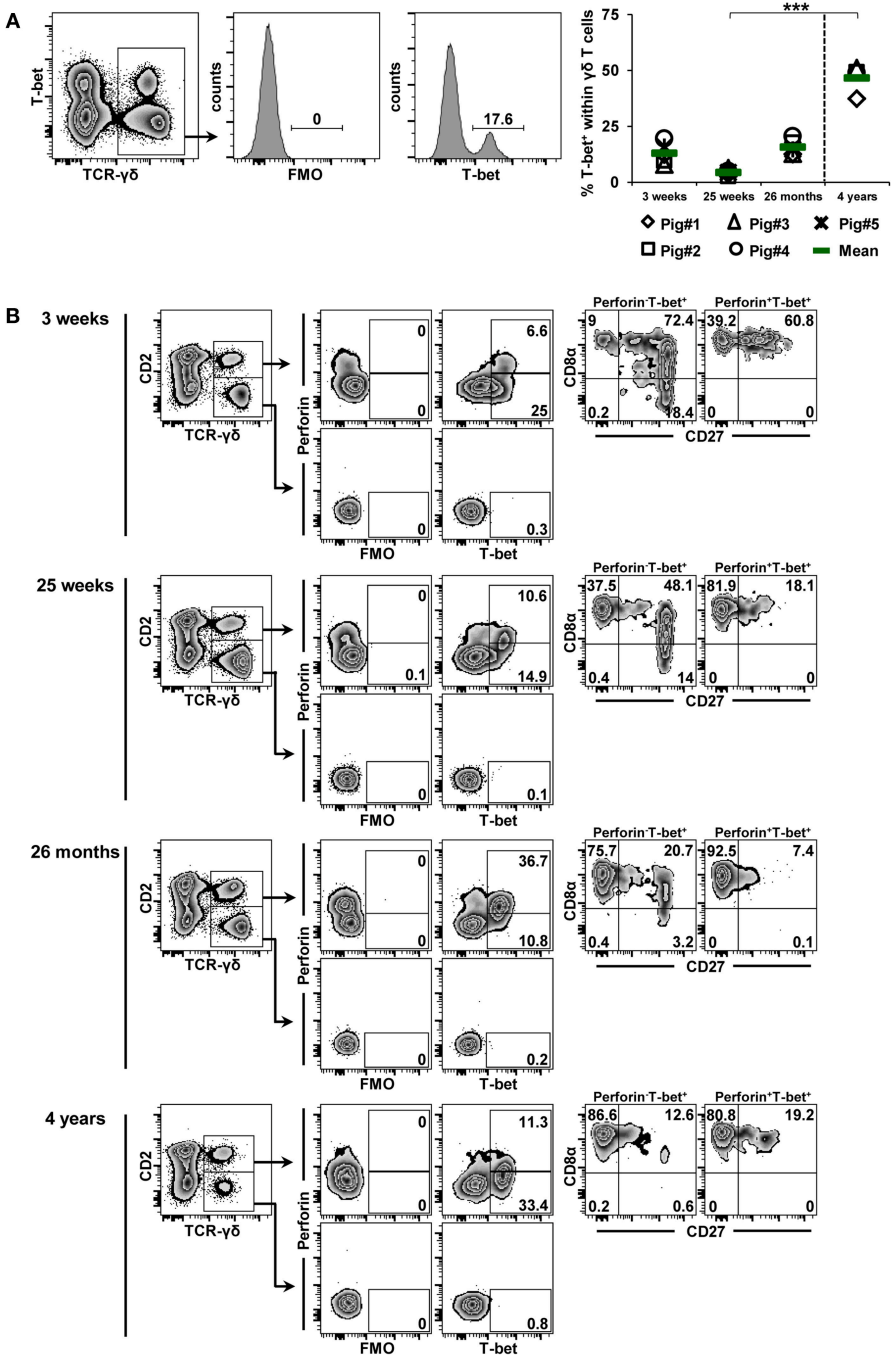
Influence of aging on the expression of T-bet in blood-derived γδ T cells. **(A)** Following exclusion of dead cells (not shown), TCR-γδ^+^ T cells were gated within live lymphocytes (zebra plot) and further subgated to analyze the expression of T-bet. The scatter diagram shows the frequency of T-bet^+^ γδ T cells within live peripheral blood lymphocytes (PBL) isolated from pigs at 3 weeks, 25 weeks, 26 months, and 4 years of age. Each symbol represents data from one individual animal (for 3 weeks, 25 weeks, and 26 months of age animals (*n* = 5) were bled repeatedly; for 4 years of age, blood was obtained from different animals (*n* = 4); change in animals is indicated by a dashed line). Green bars indicate mean values. Data were subjected to Kruskal-Wallis test with Bonferroni correction as *post-hoc* test for pairwise comparison. **(B)** γδ T cells were gated for CD2^+^ and CD2^−^ subsets and further analyzed for co-expression of perforin and T-bet. Perforin^+^ and perforin^−^ T-bet^+^ γδ T cells were then gated and investigated for expression of CD27 and CD8α. Representative data for the four investigated stages of life are shown. Numbers located in gates and quadrants indicate percentage of cells for one particular phenotype. For T-bet/perforin-defined subpopulations below 10%, CD8α/CD27 expression is shown in dot-plots, for T-bet/perforin-defined subpopulations below 2%, CD8α/CD27 expression is not shown.

**Figure 7:**
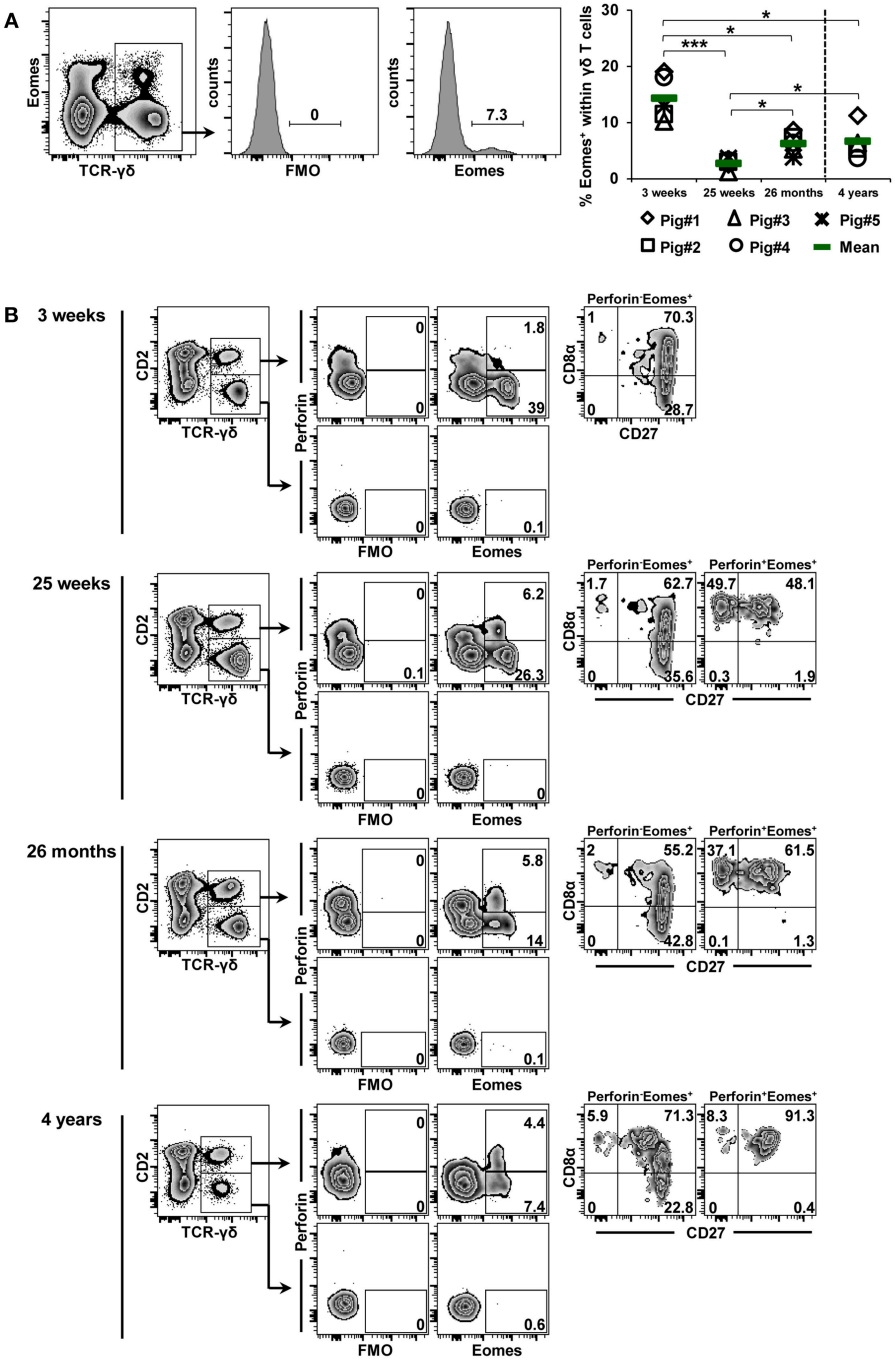
Influence of aging on the expression of Eomes in blood-derived γδ T cells. **(A)** Following exclusion of dead cells (not shown), TCR-γδ^+^ T cells were gated within live lymphocytes (zebra plot) and further subgated to analyze the expression of Eomes. The scatter diagram shows the frequency of Eomes^+^ γδ T cells within live peripheral blood lymphocytes (PBL) isolated from pigs at 3 weeks, 25 weeks, 26 months, and 4 years of age. Each symbol represents data from one individual animal (for 3 weeks, 25 weeks, and 26 months of age animals (*n* = 5) were bled repeatedly; for 4 years of age, blood was obtained from different animals (*n* = 4); change in animals is indicated by a dashed line). Green bars indicate mean values. Data were subjected to one-way ANOVA analysis with Bonferroni correction as *post-hoc* test for pairwise comparison after positive testing for normal distribution (Shapiro-Wilk test). **(B)** γδ T cells were gated for CD2^+^ and CD2^−^ subsets and further analyzed for co-expression of perforin and Eomes. Perforin^+^ and perforin^−^ T-bet^+^ γδ T cells were then gated and investigated for expression of CD27 and CD8α. Representative data for the four investigated stages of life are shown. Numbers located in gates and quadrants indicate percentage of cells for one particular phenotype. For Eomes/perforin-defined subpopulations below 10%, CD8α/CD27 expression is shown in dot-plots, for Eomes/perforin-defined subpopulations below 2%, CD8α/CD27 expression is not shown.

**Figure 8:**
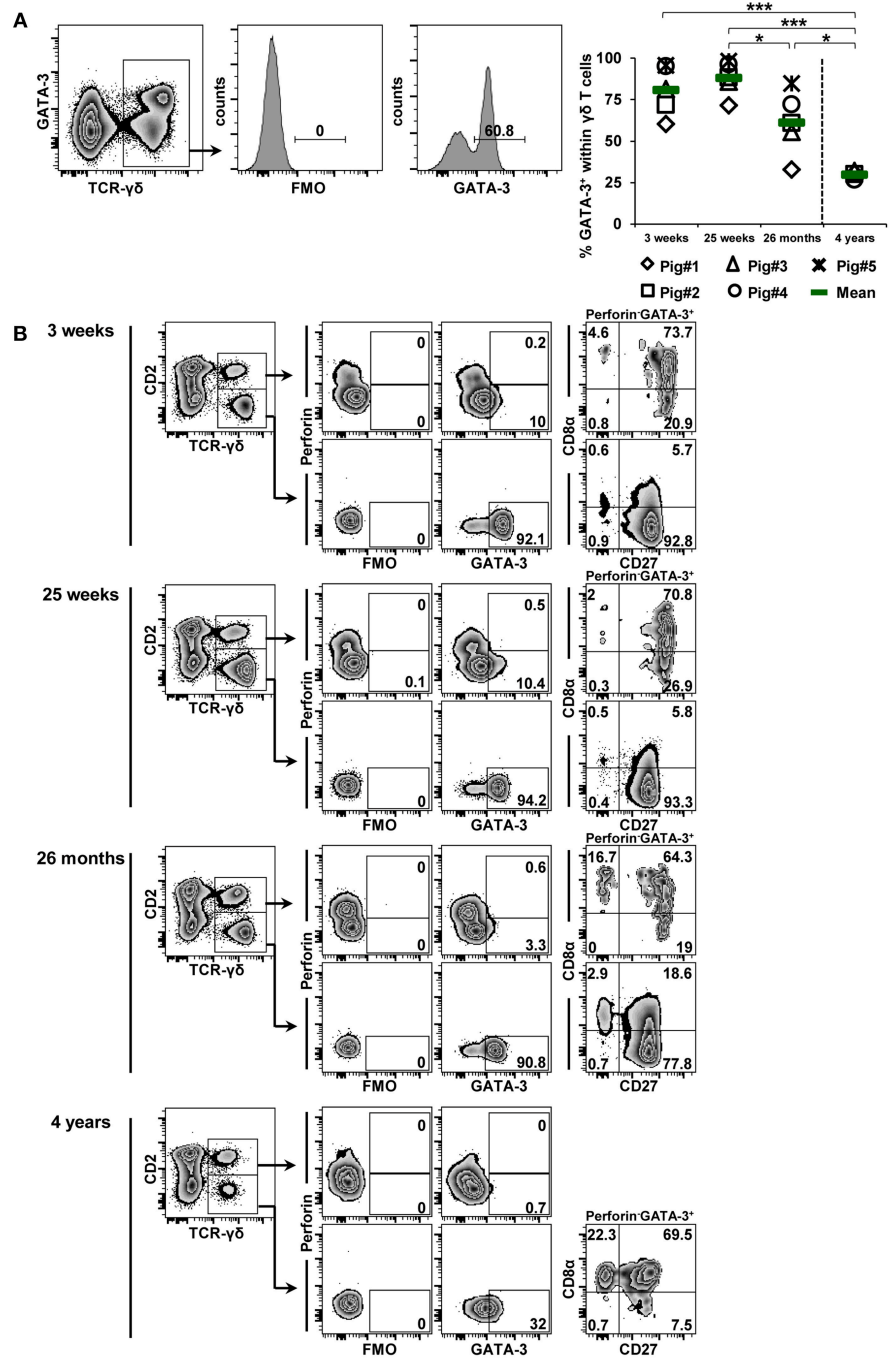
Influence of aging on the expression of GATA-3 in blood-derived γδ T cells. **(A)** Following exclusion of dead cells (not shown), TCR-γδ^+^ T cells were gated within live lymphocytes (zebra plot) and further subgated to analyze the expression of GATA-3. The scatter diagram shows the frequency of GATA-3^+^ γδ T cells within live peripheral blood lymphocytes (PBL) isolated from pigs at 3 weeks, 25 weeks, 26 months, and 4 years of age. Each symbol represents data from one individual animal (for 3 weeks, 25 weeks, and 26 months of age animals (*n* = 5) were bled repeatedly; for 4 years of age, blood was obtained from different animals (*n* = 4); change in animals is indicated by a dashed line). Green bars indicate mean values. Data were subjected to one-way ANOVA analysis with Bonferroni correction as *post-hoc* test for pairwise comparison after positive testing for normal distribution (Shapiro-Wilk test). **(B)** γδ T cells were gated for CD2^+^ and CD2^−^ subsets and further analyzed for co-expression of perforin and GATA-3. Perforin^+^ and perforin^−^ GATA-3^+^ γδ T cells were then gated and investigated for expression of CD27 and CD8α. Representative data for the four investigated stages of life are shown. Numbers located in gates and quadrants indicate percentage of cells for one particular phenotype. For GATA-3/perforin-defined subpopulations below 10%, CD8α/CD27 expression is shown in dot-plots, for GATA-3/perforin-defined subpopulations below 2%, CD8α/CD27 expression is not shown.

The data described so far were obtained from γδ T cells isolated from different organs of 7-month-old pigs (an age when pigs have just reached sexual maturity, therefore corresponding to early puberty in humans). Next, we aimed to address age-related changes in TF-expression in blood-derived γδ T cells. Accordingly, cryopreserved PBMCs from a group of five pigs isolated at 3 weeks, 25 weeks, and 26 months of age were used and compared to PBMCs isolated from four animals of approximately 4 years of age (with 4 years of age being equivalent to full adulthood in humans). By using the same labeling and gating strategy as in Figures 2–4, γδ T cells from these animals were analyzed for the expression of T-bet (Figure 6), Eomes (Figure 7) and GATA-3 (Figure 8) in combination with CD2, CD8α, CD27 and perforin. The frequencies of T-bet^+^ γδ T cells were around 13% at 3 weeks of age, declined to 4.4% at 25 weeks of age and increased afterwards to 15.7% at 26 months of age within this group of animals that were continuously bled over this period of time (Figure 6A). In contrast, T-bet^+^ γδ T cells in 4-year-old animals reached on average 46.7%, a frequency significantly different from values obtained in 25 week old pigs. The phenotype displayed by T-bet^+^ γδ T cells isolated from blood of these animals (Figure 6B) was similar to the one shown in Figure 2B. At all investigated stages of life, T-bet^+^ γδ T cells were CD2^+^ and frequently co-expressed perforin. Also, as presented already in Figure 2B, CD2^+^perforin^+^T-bet^+^ γδ T cells mainly had a CD8α^+^CD27^−^ phenotype (>80%), with the exception of cells isolated at 3 weeks of age, where the majority had a CD8α^+^CD27^+^ phenotype. At 3 and 25 weeks of age, CD2^+^perforin^−^T-bet^+^ γδ T cells were mainly CD8α^+^CD27^+^ (>48%) but at the two later stages of life, the majority of these cells had a CD8α^+^CD27^−^ phenotype (>75%).

Data for age-related changes in Eomes expression of γδ T cells are shown in Figure 7. Frequencies of Eomes-expressing γδ T cells were higher in the blood of 3-week-old piglets (mean 14.3%) than in the blood of 7-month-old pigs (Figure 3A, mean frequency of 6.8%). As the animals got older, there was a highly significant decrease from 3 to 25 weeks of age (mean 2.7%); thereafter, lower but significant increases were seen at 26 months and 4 years of age. The phenotype of Eomes^+^ γδ T cells showed some age-related changes (Figure 7B). As reported for blood-derived γδ T cells in Figure 3A, Eomes^+^ γδ T cells were CD2^+^ at all stages of life, but from 25 weeks of age onwards some Eomes^+^ γδ T cells co-expressed perforin. Somewhat different from T-bet^+^perforin^+^ γδ T cells, a considerable part of these cells had a CD8α^+^CD27^+^ phenotype (>48%). The same CD8α^+^CD27^+^ phenotype dominated among Eomes^+^perforin^−^ γδ T cells across all stages of life (>55%).

The frequency of GATA-3^+^ γδ T cells was already high in 3-week-old piglets (mean 80.8%), increased slightly toward 25 weeks of age (mean 87.9%) and then started to decline significantly (26 months: mean of 61.2%; 4 years: mean of 29.8%; Figure 8A). Similar to the observations in Figure 4B, nearly the entire CD2^−^ γδ T-cell population was GATA-3^+^ (Figure 8B), although at 3 and 25 weeks of age CD2^+^ γδ T cells also seemed to express low levels of GATA-3. The expression level of GATA-3 appeared to decrease with increasing age, this was especially noticeable at 4 years of age. Also in accordance with the phenotypes described in Figure 4B, GATA-3^+^ γδ T cells were perforin^−^ and within the CD2^−^ fraction these cells were mainly CD8α^−^CD27^+^ (> 77%). However, at 4 years of age, GATA-3^+^CD2^−^ γδ T cells had in their majority a CD8α^+^ phenotype. CD2^+^ γδ T cells with a low expression of GATA-3, present between 3 weeks and 26 months of life, mainly had a CD8α^+^CD27^+^ phenotype (> 64%).

**Figure 9:**
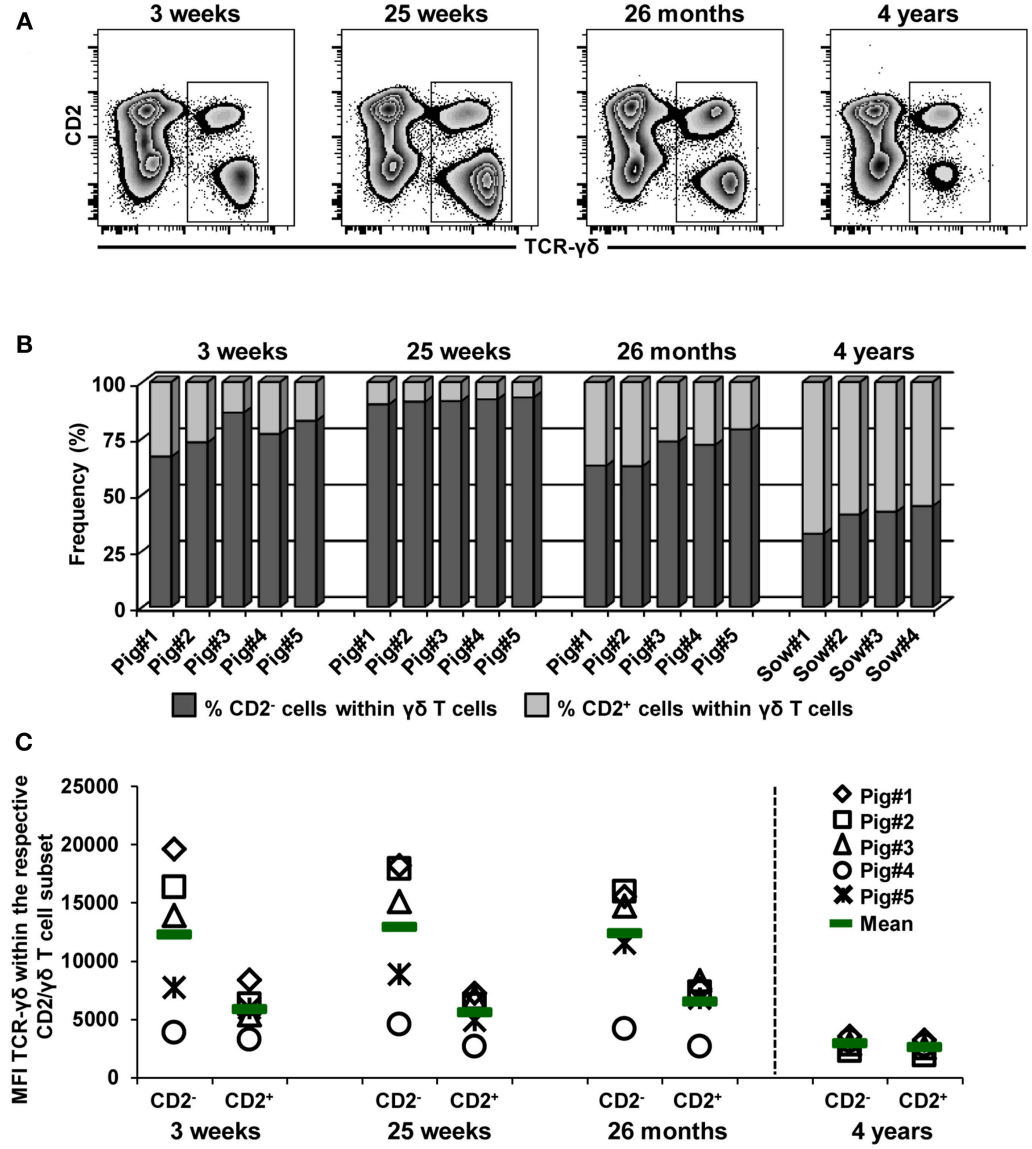
Influence of aging on the frequency of CD2^−^ and CD2^+^ γδ T cells in blood. **(A)** Live lymphocytes isolated from pigs at 3 weeks, 25 weeks, 26 months, and 4 years of age were analyzed for the expression of TCR-γδ vs. CD2 (zebra plots). Representative data from one animal per time point are shown. Total γδ T cells were gated and further subgated into CD2^−^ and CD2^+^ γδ T cells (not shown). **(B)** Stacked bar graphs indicate the frequency of CD2^−^ cells (dark gray bar) and CD2^+^ cells (light gray bar) within total γδ T cells. Data of the same five pigs are shown from 3 weeks to 26 months of age (these animals were bled repeatedly), whereas for 4 years of age, data from four different animals are presented. **(C)** Median fluorescence intensity (MFI) of TCR-γδ expression in CD2^−^ and CD2^+^ γδ T cells at different stages of life. Each symbol represents data from one individual animal (for 3 weeks, 25 weeks, and 26 months of age animals (*n* = 5) were bled repeatedly; for 4 years of age, blood was obtained from different animals (*n* = 4); change in animals is indicated by a dashed line). Green bars indicate mean values.

**Figure 10:**
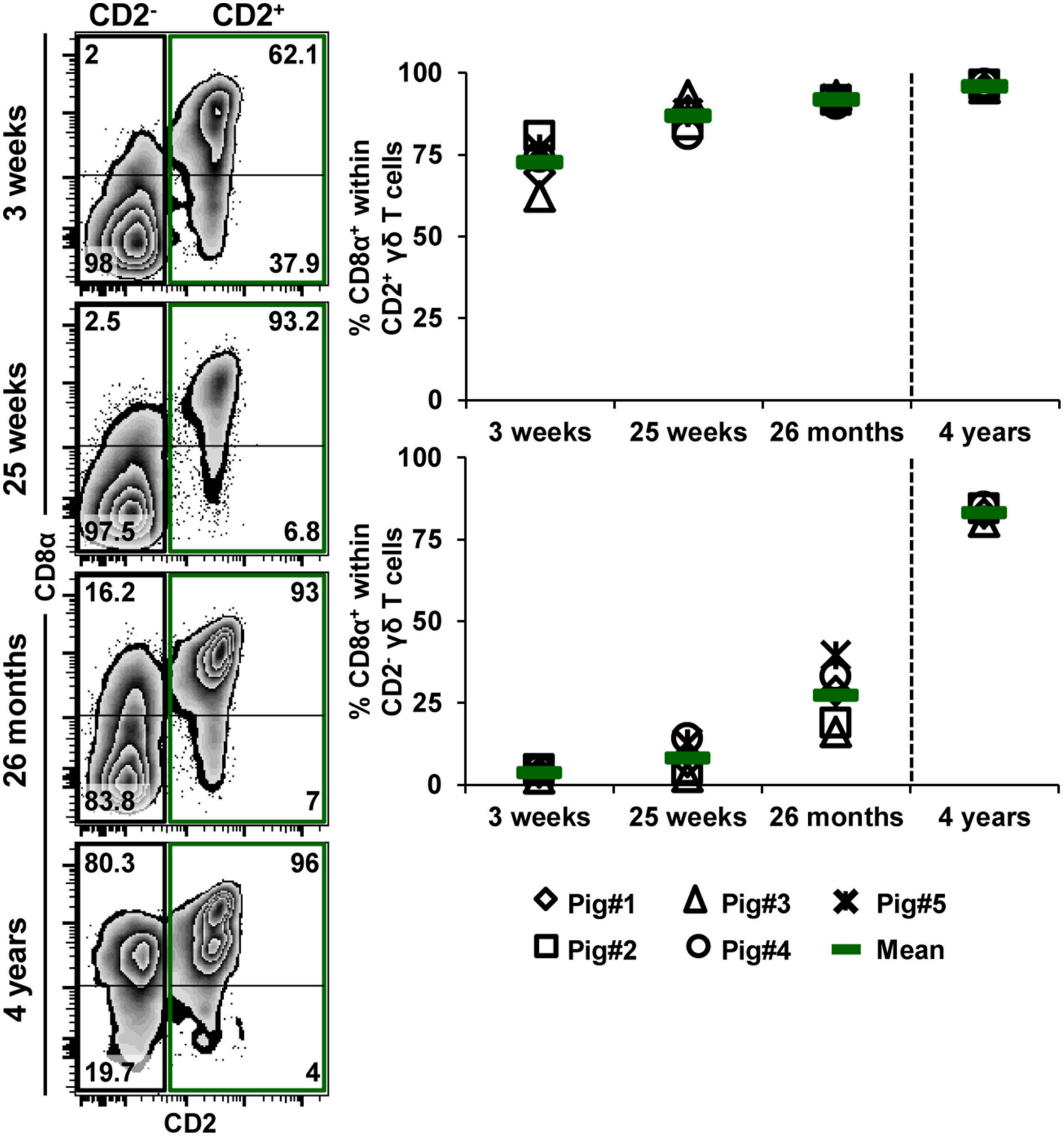
Influence of aging on CD8α expression within CD2^−^ and CD2^+^ γδ T cells. TCR-γδ^+^ cells were gated (not shown) within live lymphocytes isolated from pigs at 3 weeks, 25 weeks, 26 months, and 4 years of age and further analyzed for the expression of CD2 vs. CD8α (zebra plots, left column). Green boxes indicate gates used to identify CD2^+^ γδ T cells, black boxes indicate gates used to identify CD2^−^ γδ T cells. Numbers in the corner of each zebra plot show the percentage of cells being positive or negative for CD8α expression within CD2^−^ or CD2^+^ γδ T cells. Representative data from one animal per time point are shown. Scatter diagrams show the percentage of CD8α^+^ cells within either CD2^+^ or CD2^−^ γδ T cells. Each symbol represents data from one individual animal (for 3 weeks, 25 weeks, and 26 months of age animals (*n* = 5) were bled repeatedly; for 4 years of age, blood was obtained from different animals (*n* = 4); change in animals is indicated by a dashed line). Green bars indicate mean values.

In Figures 6–8, it also became obvious that within the γδ T cells present in the blood of 4-year-old animals CD2^−^ γδ T cells decreased at the cost of CD2^+^ γδ T cells and that CD2^−^GATA-3^+^ γδ T cells expressed CD8α. Hence, we analyzed these age-related changes in CD2 and CD8α expression in more detail (Figures 9, 10). Zebra plots in Figure 9A show the co-expression of TCR-γδ and CD2 in live lymphocytes at the different ages. In accordance with Stepanova and Sinkora (34), CD2^−^ γδ T cells expressed slightly higher levels of TCR-γδ compared to CD2^+^ γδ T cells. As shown in Figure 9B, CD2^+^ γδ T cells constituted a minor subset of the whole TCR-γδ^+^ population in blood at 3 and 25 weeks of age (mean of 22.7 and 8.3%, respectively) and there was even a decrease of this population from 3 to 25 weeks of age. However, frequencies of CD2^+^ γδ T cells were increased at 26 months (mean of 30%), and at 4 years of age mean frequencies of CD2^+^ γδ T cells against CD2^−^ γδ T cells were 60 vs. 40%, respectively. Of note, this increase in CD2^+^ γδ T cells coincided with an increase in T-bet^+^ γδ T cells (Figure 6), which, in their vast majority, had a CD2^+^ phenotype. Linked to this, the corresponding loss of CD2^−^ γδ T cells coincided with a loss of GATA-3^+^ γδ T cells (Figure 8) which were in their majority CD2^−^. In addition, expression levels of the TCR-γδ on CD2^−^ γδ T cells started to decline from 26 months onwards (Figure 9C) and nearly matched the TCR-γδ expression level on CD2^+^ cells at 4 years of age.

Additionally, we analyzed the frequency of CD8α^+^ cells within both CD2^+^ and CD2^−^ γδ T cells at the different ages (Figure 10). Zebra plots in Figure 10 show data derived from one representative animal. The frequency of CD8α^+^ cells within CD2^+^ and CD2^−^ γδ T cells continuously increased with age. However, whereas the frequencies within CD2^+^ γδ T cells increased from 72.7% at 3 weeks of age to 95.9% at 4 years of age, this gain was considerably higher for CD2^−^ γδ T cells, ranging from 3.7% at 3 weeks of age to 83.1% at 4 years of age (Figure 10, scatter diagrams).

### Analysis of IL-4 Production in γδ T Cells by Intracellular Cytokine Staining

**Figure 11:**
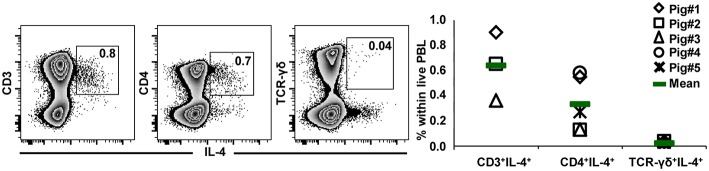
IL-4 expression in T-cell subsets after PMA/ionomycin stimulation. PBMCs isolated from the blood of 7-month-old pigs were stimulated for 4 h with PMA/ionomycin and analyzed for the expression of IL-4 in total T-cells (CD3^+^) as well as in CD4^+^ and TCR-γδ^+^ T cells. Zebra plots show live peripheral blood lymphocytes (PBL) and their expression of IL-4 vs. CD3, CD4, or TCR-γδ. CD3^+^IL-4^+^ cells, CD4^+^IL-4^+^ cells and TCR-γδ^+^IL-4^+^ cells were gated as indicated by the squares within the respective zebra plots, numbers in gates show the percentage of cells with the respective phenotype within live lymphocytes. The scatter diagram shows the percentage of CD3^+^IL-4^+^ cells, CD4^+^IL-4^+^ cells and TCR-γδ^+^IL-4^+^ cells within live PBL. Data of five individual animals (each represented by a different symbol) are shown. Green bars indicate mean values.

GATA-3 is considered to be a master regulator of Th2 differentiation, of which IL-4 production is a hallmark (17). Having observed high GATA-3 expression in the majority of CD2^−^ γδ T cells, we aimed to investigate whether these high expression levels coincided with the capacity for IL-4 production. PBMCs from 7-month-old pigs were stimulated with PMA/ionomycin and analyzed for IL-4 production by ICS. Low frequencies of CD3^+^ as well as CD4^+^ IL-4 producing T cells could be identified with this experimental set-up (Figure 11), however IL-4 producing γδ T cells were extremely rare. Hence, our results do not indicate that high expression of GATA-3 is correlated with IL-4 production in porcine γδ T cells.

### CD2^−^ γδ T Cells Express T-Bet After *in vitro* Stimulation With IL-12 and IL-18

**Figure 12:**
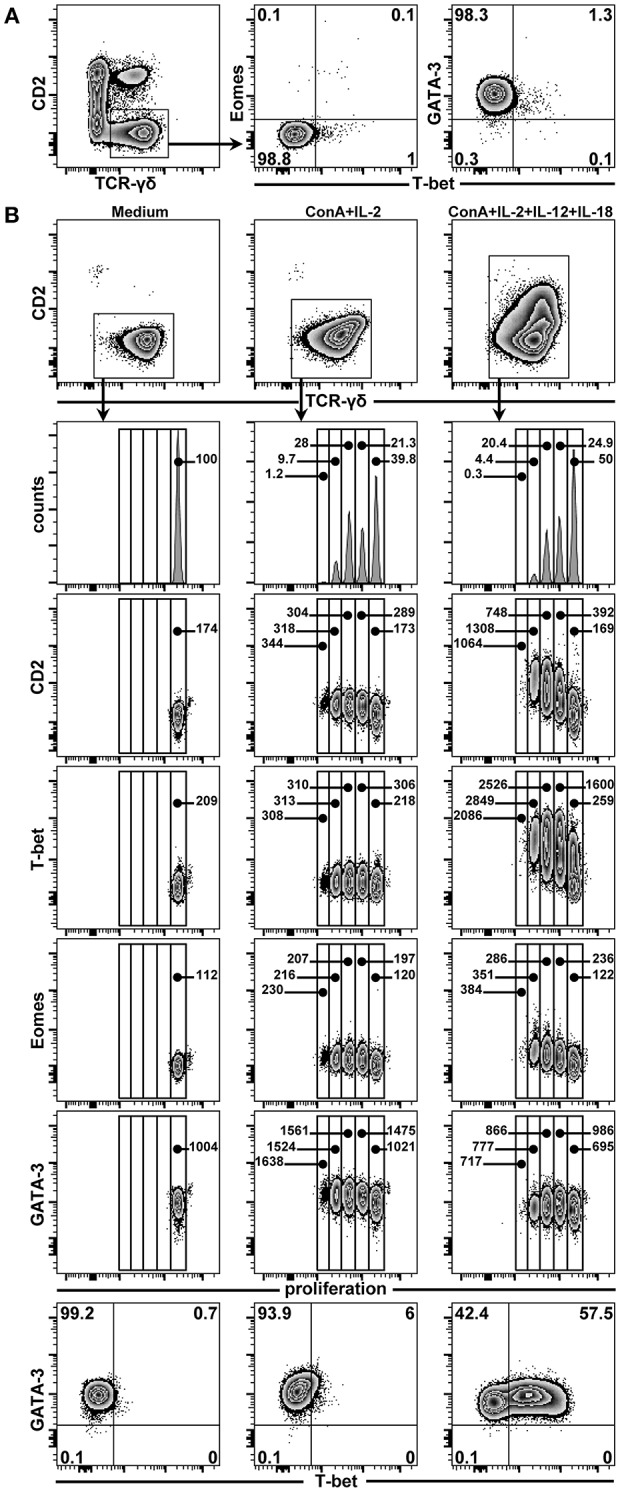
Influence of ConA and cytokine stimulation on proliferation and TF expression in CD2^−^ γδ T cells. **(A)** PBMCs isolated from blood of 7-month-old pigs were stained for TCR-γδ and CD2 expression and CD2^−^ γδ T cells were sorted by FACS (illustrated in zebra plots). Following sorting, CD2^−^ γδ T cells were analyzed for purity (not shown) and expression of T-bet in combination with either Eomes or GATA-3 (zebra plots). Numbers in the corners show percentage of cells with the respective phenotype. **(B)** In parallel, FACS-sorted CD2^−^ γδ T cells were stained with CellTrace™ Violet and cultivated in medium (first column) or stimulated with ConA + IL-2 (second column) or ConA + IL-2 + IL-12 + IL-18 (third column). After 4 days of cultivation, these cells were re-stained for TCR-γδ and CD2 expression, gated (zebra plots, top panel), and proliferation in combination with CD2 and the transcriptions factors T-bet, Eomes and GATA-3 was analyzed (histograms and subsequent zebra plots). Additionally, expression of T-bet vs. GATA-3 is shown for each stimulation condition (last row). Numbers in the histograms give the percentage of cells in each generation, as determined by dilution of the violet proliferation dye. Numbers in zebra plots give the median fluorescence intensity for CD2, T-bet, Eomes, and GATA-3 (third to fourth row of zebra plots, respectively) of cells in each generation. Numbers in the corners of zebra plots at the bottom show percentage of cells with the respective phenotype. The data shown are representative of three experiments with PBMCs from three different pigs.

In a previous study, we could show that CD2^−^ γδ T cells can convert into CD2^+^ γδ T cells following *in vitro* stimulation with a combination of ConA, IL-2, IL-12, and IL-18 (4). We now aimed to investigate whether this switch in the CD2 phenotype is also accompanied by a change in TF expression from GATA-3 to T-bet and potentially Eomes, since this was the dominant TF phenotype in CD2^+^ γδ T cells (see above). Therefore, PBMCs, FACS-sorted γδ T cells and FACS-sorted CD2^−^ γδ T cells from 7-month-old pigs were stained with violet proliferation dye and cultivated for 4 days with ConA and the aforementioned cytokines. Additionally, supernatants of such microcultures were collected and tested for IFN-γ and IL-4 production after 4 days of stimulation. Following sorting, but prior to cultivation, the expression of TFs was analyzed on sorted CD2^−^ γδ T cells (Figure 12A). In accordance with previous phenotyping experiments of total γδ T cells, these sorted CD2^−^ γδ T cells expressed GATA-3 (~98% cells in representative experiment shown), but neither T-bet nor Eomes. After 4 days of cultivation, proliferation as well as CD2 and TF expression was assessed by FCM (Figure 12B). Sorted and violet-stained CD2^−^ γδ T cells cultivated in medium neither proliferated nor changed TF expression (Figure 12B, first column). In the presence of ConA and IL-2, proliferation was induced, however, no changes in TF expression were detected (Figure 12B, second column). Addition of IL-12 and IL-18 to ConA and IL-2, both polarizing cytokines of the Th1 response (35–37), induced the proliferation of cells, *de novo* CD2 expression in proliferating γδ T cells (increase in CD2 MFI from 169 in non-poliferating γδ T cells to 1308 in third generation of proliferating γδ T cells) as well as up-regulation of T-bet (increase in T-bet MFI from 259 in non-poliferating γδ T cells to 2,849 in third generation of proliferating γδ T cells Figure 12B, third column). In contrast, Eomes expression levels showed only minor increases (increase MFI from 122 in non-poliferating γδ T cells to 351 in third generation of proliferating γδ T cells) and stimulation of cells through Th1-polarizing cytokines did not induce a downregulation of GATA-3 expression (MFI varied between 695 and 986). Similar results were obtained for total sorted γδ T cells and PBMCs in parallel cultivation experiments, although in cultures consisting of PBMCs a slight upregulation of T-bet on γδ T cells was seen already in the presence of ConA and IL-2 only (Supplementary Figure 2), which may be a result of other cells producing type-1 polarizing cytokines in such bulk cultures following ConA and IL-2 stimulation.

With regard to IFN-γ production, combined stimulation with ConA and the cytokine cocktail led to production of IFN-γ in the cell culture supernatants of PBMCs, total sorted γδ T cells and sorted CD2^−^ γδ T cells above the detection level of the FMIA assay, albeit some degree of variation between subsets and pigs was detected (Supplementary Figure 3). In contrast, IL-4 production in microcultures with sorted γδ T cells was always below the detection limit, with the exception of one pig, where very low levels (10 pg/mL) of IL-4 were detected in the supernatant of ConA + IL-2 + IL-12 + IL-18 stimulated CD2^−^ γδ T cells (data not shown). This seems to indicate that the observed upregulation of T-bet and CD2 in sorted CD2^−^ γδ T cells was accompanied by a gain of function for IFN-γ production.

## Discussion

In humans and mice, GATA-3 has been shown to be essential for T-cell development and also for the functional polarization of T cells, whereas T-bet and Eomes seem to be involved only in the latter (38–40). Recently, our group described the role of T-bet, Eomes and GATA-3 in porcine TCR-αβ^+^ lymphocytes (28). However, data on the expression of these three TFs in porcine γδ T cells are still missing and hence, we analyzed their expression in γδ T cells isolated from the thymus, blood, spleen, mediastinal lymph node, and lung tissue of healthy pigs by flow cytometry.

In various mouse models, it could be shown that GATA-3 is essential for thymocyte development (18, 41), including γδ thymocytes (19). We found that ~90% of porcine γδ^+^ thymocytes express GATA-3 which seems to confirm the relevance of this TF for the development of γδ T cells in swine.

More surprising was the finding that a substantial proportion of γδ T cells expressed high levels of GATA-3 in all investigated secondary lymphoid organs as well as in lung tissue and these levels were even higher than found for CD4^+^ T cells in a previous study from our group (28). This is in contrast to studies in mice, where neither GATA-3 mRNA nor protein was detected in naïve (CD44^low^CD62L^high^) γδ T cells (16). Also for activated (CD44^high^) γδ T cells, in that study, GATA-3 could not be found on the protein level, although a weak expression was identified at mRNA level. Our data also revealed a positive correlation of GATA-3 expression with a distinct phenotype: CD2^−^perforin^−^CD8α^dim/−^CD27^+^. In the pig, naïve CD4^+^ T cells have a CD8α^−^CD27^+^ phenotype (42, 43) and similarly, naïve porcine CD8^+^ T cells are considered to be CD27^+^perforin^−^ (5). This may suggest that the identified GATA-3^+^CD8α^dim/−^CD27^+^perforin^−^ γδ T cells are naïve. The relevance of CD2 expression for the differentiation of porcine γδ T cells is less clear. Early studies indicated an enrichment of CD2^+^ γδ T cells in lymphoid organs compared to the blood (44). More recent studies from Stepanova and Sinkora (34) suggested that CD2^−^ and CD2^+^ γδ T cells are two separate cell lineages that become already established in the thymus. Indeed, CD2^−^ γδ T cells seem to be a peculiarity of pigs, sheep (45), cattle (46), and chicken (47), all of which are considered as “γδ-high” species (48). It is worth noting that also in human PBL CD2^−^ T cells are present in very low frequencies (<0.1 % within total CD3^+^ T cells) of which approximately 50% have a TCR-γδ (49) and express GATA-3 (50). These cells have mainly a CD4^+^ phenotype and a high capacity for CD3-mediated proliferation, which led to the assumption that these cells may exhibit extrathymic maturation and contribute to the maintenance of the peripheral naïve T-cell pool (49). Related to this, more recently a scarce population of CD4^+^Vδ1^+^ γδ T cells in human blood has been investigated in more detail, revealing that these cells display features of T-cell progenitors, among them GATA-3 expression. It was shown that these cells can dedifferentiate into αβ T cells (51). However, the vast majority of extrathymic porcine γδ T cells is CD4^−^ (52). In addition, CD2^−^ γδ thymocytes appear to develop separately from CD2^+^CD4^+^ γδ thymocytes, and the latter seem not to get exported to the periphery (31). Therefore, our data corroborate the notion that CD2^−^ γδ T cells are a developmentally distinct γδ T-cell subpopulation which is already established in the thymus and which retains a high GATA-3 expression in the periphery.

T-bet^+^ cells were scarce within γδ T cells isolated from mediastinal lymph nodes but more frequent within TCR-γδ^+^ T cells from blood, spleen and lung tissue. Most of these cells displayed the phenotype CD2^+^perforin^+^CD8α^+^CD27^−^, which, together with organ localization, seems to indicate an effector or terminally differentiated phenotype in combination with a type-1 polarization. Similarly, in murine γδ T cells with an effector phenotype (CD44^high^), T-bet mRNA and protein were found (16). Within blood-derived human γδ T cells, a substantial T-bet^+^ subpopulation (~60%) has been described (14), but no information on the differentiation-related phenotype of these cells was provided.

The frequency of Eomes-expressing γδ T cells in the periphery varied from moderate to high, especially in the spleen where up to 62% of γδ T cells expressed Eomes. This is in accordance with a recent study in mice (15), which found the highest frequency of Eomes^+^ γδ T cells also in the spleen. In our study, the predominant phenotype of Eomes^+^ γδ T cells was CD2^+^CD8α^+^CD27^+^perforin^−^, which may indicate that these cells are less differentiated than the T-bet^+^CD2^+^CD8α^+^CD27^−^perforin^+^ γδ T cells we identified. For mice, one study reported the expression of Eomes already in γδ T cells with a naïve phenotype (CD44^low^CD62L^high^; 13). However, the more recent study by Lino et al. (15) indicated that murine Eomes^+^ γδ T cells are strongly enriched in CD44^+^Ly6C^+^ γδ T cells and have a higher capacity for IFN-γ production compared to their Eomes^−^ counterparts, which the authors interpreted as an activated phenotype. In accordance with our findings, these authors also reported that the vast majority of murine Eomes^+^ γδ T cells were CD27^+^ (15).

We also found a co-expression of T-bet and Eomes in γδ T cells from blood, spleen and lung tissue. Similar findings have been reported for γδ T cells in mice and humans (13–15). However, for mice a mainly linear co-expression has been described (15), and in humans, Eomes^+^ γδ T cells showed a T-bet^high^ phenotype (14), whereas we found that porcine Eomes^+^ γδ T cells have somewhat lower or equal T-bet expression levels than T-bet^+^Eomes^−^ γδ T cells (Figure 5C). The functional relevance of these different co-expression patterns between species remains an open question.

Overall, the relatively high abundance of T-bet^+^CD2^+^perforin^+^CD8α^+^CD27^−^ γδ T cells in lung tissue may indicate that these cells represent late effector memory cells with cytolytic capacity in this non-lymphatic organ. In contrast, the high frequency of Eomes^+^CD2^+^perforin^−^CD8α^+^CD27^+^ γδ T cells in the spleen coincided frequently with T-bet co-expression (Figures 5A,B) and these cells may represent more long-lived, less terminally differentiated γδ T cells which preferably home to this organ. However, this is highly speculative, and needs to be corroborated by further functional studies. Also, it should be noted that although the lack of CD27 expression for CD4^+^ and CD8^+^ T cells also in the pig seems to identify late effector or effector memory T cells (5, 27), to our knowledge this has not been demonstrated for porcine γδ T cells so far. GATA-3^+^ γδ T cells were prominent in all investigated organs, however their CD2^−^ phenotype suggests that they have no immediate connection with T-bet^+^ or Eomes^+^ γδ T cells and may indeed represent a functionally different subset of porcine γδ T cells (see also below).

In mice, it was shown that both Eomes and T-bet are involved in IFN-γ production by γδ T cells (13). Moreover, it has been shown that murine splenic γδ T cells have an intrinsic type-1 polarization, even in the presence of IL-4 and absence of IL-12, due to T-bet expression following TCR-stimulation, and that GATA-3 failed to counterbalance IFN-γ production (16, 53). We sorted porcine GATA-3^+^CD2^−^ γδ T cells from blood and stimulated them in the presence of ConA+IL-2+IL-12+IL-18. While non-proliferating cells stayed negative for CD2 and T-bet, four days post stimulation all proliferating cells expressed T-bet and CD2, the latter in accordance with previous data from our group (4). Also, IFN-γ was found in the supernatant of these cells but no major changes in Eomes expression were detected, suggesting that mainly T-bet is involved in IFN-γ production in porcine γδ T cells under these conditions. This outcome is in agreement with published data from Barros-Martins et al. (54), who showed that Eomes is fully dispensable for IFN-γ production in murine CD27^+^ γδ T cells. Of note, in our *in vitro* experiments with GATA-3^+^CD2^−^ γδ T cells, stimulation with ConA+IL-2+IL-12+IL-18 did not negatively affect GATA-3 expression, which might be explained by a constant stimulation of the TCR-signaling pathway via the combined action of ConA, IL-12 and IL-18, since TCR-stimulation has been shown to upregulate GATA-3 expression (33).

Our studies on age-related changes in phenotype and TF expression in porcine γδ T cells revealed an increase of T-bet^+^CD2^+^ cells in older animals at the expense of GATA-3^+^CD2^−^ γδ T cells. Moreover, for both CD2^−^ and CD2^+^ γδ T cells, a gradual increase in the frequency of CD8α^+^ cells with increasing age was observed. The functional relevance of these phenotypic changes is currently not clear. A constant increase in CD8α expressing cells with age is also well described for porcine CD4^+^ T cells (28, 52) and it was suggested that this denotes a constant rise in antigen-experienced CD4^+^ T cells. Whether the same applies for porcine γδ T cells needs to be elucidated by further investigations. The change in the ratio of T-bet^+^CD2^+^ to GATA-3^+^CD2^−^ γδ T cells again may indicate that CD2^+^ and CD2^−^ γδ T cells are separate T-cell lineages in swine. However, a further interpretation of this current notion requires a better understanding of the function of these cells. Our latest unpublished findings show that CD28 expression is restricted to a subset of CD2^+^ γδ T cells, whereas all CD2^−^ γδ T cells lack CD28 expression. Given the high relevance of CD28 in TCR-mediated activation, this may indicate that CD2^−^ γδ T cells in pigs rely on TCR-independent mechanisms for activation. This speculation is also supported by the exclusive expression of swine workshop cluster 5 in CD2^−^ γδ T cells (3, 4), which most probably is a member of the scavenger receptor cysteine-rich superfamily, for which a function as pattern recognition receptor has been described in bovine γδ T cells (55, 56). Therefore, one hypothesis emerging from these observations might be that GATA-3^+^CD2^−^ γδ T cells constitute indeed a separate γδ T cell lineage that persists in the periphery after release from the thymus up to a high age of individual animals, and recognize antigen in a TCR-independent manner. However, the functional capacities of these cells are not known yet and it should be noted that there seems to be no corresponding population of γδ T cells in rodents or humans, at least not in such high numbers (see above).

In summary, our studies on the expression of T-bet, Eomes and GATA-3 in porcine γδ T cells reveal the assignment of these transcription factors to distinct subsets and phenotypes of γδ T-cells. This paves the way for new hypotheses on the functions of these different subsets that should help to clarify the general role of γδ T cells in swine, which are a prominent but still enigmatic T-cell population in this species.

## Author Contributions

IR-G, TK, AS, and WG were responsible for the design of the study and conceived experiments. IR-G performed experiments and analyzed data. ST, MS, SH, JM, and KM carried out laboratory work and experiments. LR and AL performed multiplex fluorescent microsphere immunoassays. IR-G and WG wrote the manuscript. All authors read and approved the final manuscript.

### Conflict of Interest Statement

The authors declare that the research was conducted in the absence of any commercial or financial relationships that could be construed as a potential conflict of interest.
