# Supplementary material for: Expression of T-Bet, Eomesodermin, and GATA-3 Correlates With Distinct Phenotypes and Functional Properties in Porcine γδ T Cells
Source: Front Immunol. 2019 Mar 11;10:396. doi: 10.3389/fimmu.2019.00396 (PMC6421308; doi:10.3389/fimmu.2019.00396)
Supplement: Supplementary file 1 [file Data_Sheet_1.docx]

Supplementary Material

Expression of T-bet, Eomesodermin and GATA-3 correlates with distinct phenotypes and functional properties in porcine γδ T cells

Irene M. Rodríguez-Gómez, Stephanie C. Talker, Tobias Käser, Maria Stadler, Lisa Reiter, Andrea Ladinig, Jemma V. Milburn, Sabine E. Hammer, Kerstin H. Mair, Armin Saalmüller, Wilhelm Gerner*

*** Correspondence:** Wilhelm Gerner: [wilhelm.gerner@vetmeduni.ac.at](mailto:wilhelm.gerner@vetmeduni.ac.at)

## Supplementary Figures

**Supplementary Figure 1.** I**nfluence of TCR-γδ labeling by monoclonal antibodies on GATA-3 expression**. PBMCs were labeled with mAbs against CD4, CD8β, CD16 and CD172a. Following fixation and permeabilization, cells were further incubated with mAbs against CD79α and GATA-3. All mAbs, with the exception of the anti-GATA-3 mAb, were either directly conjugated with Alexa647 or labeled via an isotype-specific Alexa647-conjugated secondary antibody, resulting in a bulk staining of the aforementioned molecules. Lymphocytes were gated based on light scatter properties and following exclusion of dead cells (not shown), bulk staining and GATA-3 expression were analyzed. Samples without GATA-3 specific mAbs served as FMO controls (left panel). Further samples were labeled in the same way but additionally stained with mAbs against CD2 and TCR-γδ (right panel). GATA-3^-^ and GATA-3^+^, non-bulk-stained cells were gated and analyzed for expression of TCR-γδ and CD2 (right panel). Numbers in the gates and quadrants show percentage of cells with the respective phenotype. Data are representative for staining experiments with PBMCs from four different seven-month-old pigs.

**Supplementary Figure 2. Influence of ConA and cytokine stimulation on proliferation and TF expression in total γδ T cells and PBMCs.** Total γδ T cells sorted by FACS (left panel) and PBMCs (right panel), both obtained from the blood of seven-month-old pigs, were stained with CellTrace^TM^ Violet. Then, each cell fraction was cultivated in medium (first column) or stimulated with ConA + IL-2 (second column) or ConA + IL-2 + IL-12 + IL-18 (third column). After four days of cultivation, proliferation in combination with expression of CD2, T-bet, Eomes and GATA-3 was analyzed for each cell fraction and stimulation condition (histograms and zebra plots). Numbers in the histograms (top panel) give the percentage of cells in each generation, as determined by dilution of the violet proliferation dye. Numbers in zebra plots give the median fluorescence intensity for CD2, T-bet, Eomes and GATA-3 (top to bottom panels, respectively) of cells in each generation.

**Supplementary Figure 3. IFN-γ production in γδ T cells and PBMCs after *in vitro* stimulation.** Supernatants from CellTrace^TM^ Violet-stained PBMC, FACS-sorted total γδ T cells and FACS-sorted CD2^-^ γδ T cells cultivated in medium, ConA + IL-2 or ConA + IL-2 + IL-12 + IL-18 were analyzed for IFN-γ expression by multiplex fluorescent microsphere immunoassay. Bar graphs show the amount of IFN-γ production in cell culture supernatants of each cell fraction and stimulation condition used (x-axis) for cells derived from three individual pigs.
